# A Cortico-Basal Ganglia-Thalamic Network Model Linking Intermittent Postural Control to Sway-Related Beta-Band Oscillations

**DOI:** 10.1523/ENEURO.0283-26.2026

**Published:** 2026-09-18

**Authors:** Shota Tsugaya, Akihiro Nakamura, Taishin Nomura

**Affiliations:** ^1^Graduate School of Engineering Science, The University of Osaka, Osaka 560-8531, Japan; ^2^Graduate School of Informatics, Kyoto University, Kyoto 606-8501, Japan

**Keywords:** action selection, basal ganglia, beta oscillation, decision-making, Parkinson’s disease, postural control

## Abstract

Electroencephalographic (EEG) studies of human quiet stance demonstrate beta-band event-related desynchronization (beta-ERD) during the micro-fall phase of postural sway, followed by event-related synchronization (beta-ERS; post-movement beta rebound) during the micro-recovery phase. These modulations correlate with intermittent ankle muscle inactivation that exploits the stable manifolds of an unstable upright equilibrium; however, how such sway-related beta dynamics arise within closed-loop brain–body interactions remains unclear. Here, we investigated a possible circuit-level account of these dynamics using an embodied spiking neural network model of the cortico-basal ganglia-thalamic (CBGT) circuitry integrated with an inverted pendulum. In this closed-loop system, continuous sensory feedback is integrated into the striatum, while the motor cortex executes decisions via drift-diffusion-like population competition, where decision time (DT) represents the intermittent control-off period. We demonstrate that simulated cortical LFPs exhibit characteristic sway-phase-locked beta-ERD and beta-ERS when corticostriatal synaptic weights are functionally balanced to implement intermittent control. Conversely, a forced-choice continuous-like regime that ceaselessly generates feedback torque fails to replicate these modulations, sustaining flat network states devoid of control-off periods (DT). Structural dissections show that, within the model, disrupting bidirectional thalamocortical loops or the GPe–STN circuit abolishes sway-phase-locked beta modulation, despite continuous sensory drive. Our findings provide a computational account linking sway-phase-locked beta activity to intermittent motor selection within the proposed CBGT framework. This closed-loop modeling framework offers a testable candidate account for how alterations in brain–body dynamics may jointly affect behavioral intermittency and beta-band modulation, with potential relevance to postural impairments in Parkinson's disease.

## Significance Statement

Intermittent motor commands during quiet standing are accompanied by phase-specific cortical beta-band modulations, but how these neural dynamics may relate to intermittent postural control remains unclear. Here, using a closed-loop model integrating spiking cortico-basal ganglia-thalamic (CBGT) circuitry with body dynamics, we show that beta modulations arise when the model implements intermittent control. Within the model, these modulations are abolished by disrupting thalamocortical or GPe–STN interactions and are absent in a forced-choice continuous-like regime. These findings provide a computational account linking intermittent postural control to sway-related beta dynamics and identify circuit interactions sufficient for their emergence within the proposed CBGT framework. The model offers testable predictions for investigating how brain–body interactions shape beta dynamics in healthy and impaired postural control.

## Introduction

Electroencephalography (EEG) provides a crucial window into the neural computations underlying motor control. Frequency-specific modulations of ongoing cortical activity, particularly within the beta-band (13–30 Hz), are closely linked to motor preparation and execution (Pfurtscheller and Lopes da Silva, 1999). Specifically, event-related desynchronization (ERD) occurs during action preparation and execution, followed by event-related synchronization (ERS), or “beta rebound,” during movement cessation or status quo maintenance (Pfurtscheller and Lopes da Silva, 1999; Stančák et al., 2000; Neuper and Pfurtscheller, 2001; Steriade, 2006). These dynamics carry profound clinical significance; patients with Parkinson's disease exhibit pathological beta oscillations and altered modulation patterns that directly correlate with severe motor deficits (Brown et al., 2001; Jenkinson and Brown, 2011; Moisello et al., 2015; Wu et al., 2019).

The cortico-basal ganglia-thalamic (CBGT) circuit is considered the primary neuroanatomical substrate for generating these movement-related beta rhythms (Roopun et al., 2008, 2010; Singh, 2018; Vinding et al., 2019). Animal models suggest that glutamatergic inputs to the subthalamic nucleus (STN) and its reciprocal interactions with the globus pallidus (GP) play a critical role in rhythmic amplification (Tachibana et al., 2011). Despite extensive research, the exact mechanisms governing beta modulation remain controversial, partly due to the limited resolution of scalp EEG (Cohen, 2017; Ouyang et al., 2022). While computational models have variously attributed beta oscillations to the STN–GPe circuit or the striatum (Bevan et al., 2002; Terman et al., 2002; Holgado et al., 2010; McCarthy et al., 2011; Pavlides et al., 2015; Ouyang et al., 2022), recent work shows that strong pallidostriatal loops drive low-beta (10–15 Hz) oscillations, whereas strong recurrent STN–GPe or intra-GPe connections drive gamma (>40 Hz) oscillations (Lindi et al., 2024). Crucially, most conventional models focus solely on isolated neural dynamics, neglecting bidirectional, closed-loop interactions with the musculoskeletal system.

Human quiet stance offers a unique and powerful paradigm to study this bidirectional interaction. Recent empirical findings show that postural sway is accompanied by transient cortical beta-ERD and beta-ERS, corresponding to the micro-fall and micro-recovery phases, respectively (Nakamura et al., 2023). These cortical modulations coincide with discrete bursts of calf muscle activity (Loram et al., 2005a), suggesting that cortical interventions in automatic postural control operate via discrete, intermittent commands (Asai et al., 2009; Gawthrop et al., 2011; Nomura et al., 2022) rather than continuous modulations (Winter et al., 1998). This supports an “intermittent control” strategy that dynamically exploits the stable manifold of an unstable upright equilibrium (Bottaro et al., 2008; Asai et al., 2009). The involvement of the basal ganglia in this process is corroborated by recent work demonstrating that an intermittent control strategy—defined by switching boundaries in the state space—can be effectively acquired through reinforcement learning (Takazawa et al., 2024). Because the CBGT circuit plays a central role in reinforcement learning, postural stability may involve optimization of synaptic efficacies within this loop to facilitate intermittent motor selection.

In this study, we examined how sway-related beta oscillations may arise within a closed-loop model of intermittent postural control using a spiking neural network (SNN) model of the CBGT circuitry. Crucially, the model stabilizes a physical inverted pendulum in real time, while simultaneously simulating EEG via the summation of cortical postsynaptic currents (PSCs). By systematically adjusting the synaptic weights of corticostriatal projections—key targets for motor learning and dopamine depletion (Cataldi et al., 2022)—we demonstrate that simulated EEG exhibits realistic beta-ERD and beta-ERS when the network is functionally tuned for intermittent control operated under small feedback gains. Conversely, a forced-choice continuous-like regime operated continuously under large feedback gains fails to produce these characteristic cortical modulations. While Lindi et al. (2024) examined the generation of parkinsonian beta oscillations within a disembodied network, our closed-loop neuro-biomechanical model enables us to examine how beta modulation may emerge from interactions between CBGT network dynamics and physical postural dynamics. Ultimately, our results provide a novel computational link between the functional tuning of the basal ganglia and sway-related cortical beta dynamics associated with intermittent postural control.

## Materials and Methods

### Physiological foundations and abstractions of CBGT circuit topology

We developed a SNN model of the CBGT circuit by adapting a previous neural mass model designed for simulating finger tapping (Ursino et al., 2020). In our framework, we replaced each neural mass component with a population of spiking neurons and integrated a physical model of an inverted pendulum, which was absent in the original Ursino model. Within this closed-loop architecture, sensory information regarding the pendulum's kinematic state is represented by neuronal firing in the sensory cortex and fed back to the striatum. The motor cortex, positioned at the output terminal of the CBGT loop, decides between dorsiflexion and plantarflexion via competition between antagonistic motoneuronal populations. This competitive process follows drift-diffusion dynamics (Ratcliff et al., 2016), where the decision time (DT) represents the “control-off period” of the intermittent controller. Preliminary parameter exploration indicated that the synaptic weights (W) projecting from the sensory cortex to the striatum are the critical determinants governing the degree of competition between the two cortical motoneuron populations; we therefore tuned these parameters as detailed below.

We implemented variants of the CBGT model, including those corresponding to an intermittent control model operated under small feedback gains and a forced-choice continuous-like regime operated under large feedback gains, where typical values of small and large gains were employed, respectively, based on the previous study that assimilates postural sway data from healthy people and patients with Parkinson's disease with severe postural impairment (Suzuki et al., 2020). Using these models, we conducted two distinct computational experiments. Experiment 1 involved a postural control task to stabilize the inverted pendulum and reconstruct EEG dynamics during quiet stance. This experiment was designed to compare movement-related beta modulations between the intermittent CBGT regime and the forced-choice continuous-like regime. Experiment 2 was conducted to identify network interactions contributing to the generation of movement-related beta modulations within the model. In this experiment, the transmission line conveying motor commands from the cortex to the pendulum was disconnected, and the pendulum was moved passively along a prescribed, periodic trajectory independent of the CBGT cortical output. This setup allowed us to examine how model-generated EEG dynamics are influenced by state-dependent sensory signals under various network conditions, without confounding the analysis with the requirements of postural stability.

### Inverted pendulum model

To simulate the human body during quiet stance, we employed an inverted pendulum model, which simplifies a previously validated postural model (Asai et al., 2009). The inverted pendulum rotates in the sagittal plane around a pinned joint corresponding to the ankle. The dynamics of the system are governed by the following equation of motion:

$$\begin{array}{c} I\dot{\omega }=mgh\theta -k\theta -b\omega +T_{\mathrm{act}}+\sigma \xi \end{array},$$

where θ and ω are the tilt angle and angular velocity of the pendulum, respectively; $I\;(60\;\mathrm{kg}\;m^{2})$ is the moment of inertia of the pendulum around the joint; m(60kg) is the pendulum mass; $g\;(9.81\;m/s^{2})$ is the gravitational acceleration; and h(1.0m) is the distance from the joint to the center of mass. The parameters k(471Nm/rad) and b(4.0Nms/rad) represent the passive torsional elasticity and the viscosity of the joint, respectively, derived from empirical identifications (Loram and Lakie, 2002; Casadio et al., 2005). The term σξ represents zero-mean Gaussian white noise with a standard deviation of σ. The term $T_{\mathrm{act}}$ represents the active feedback torque selected by the motor cortex of the CBGT model at each simulation time step as from three possible actions: $\left\{-|P\theta_{\Delta }+D\omega_{\Delta }|,\;0,\;|P\theta_{\Delta }+D\omega_{\Delta }|\right\}$, where $\theta_{\Delta }=\theta (t-\Delta )$ and $\omega_{\Delta }=\omega (t-\Delta )\;$ represent the time-delayed state of the pendulum, reflecting a neural transmission delay of Δ=0.2s. The parameters P and D are the proportional and derivative feedback gains to generate backward torque $\left(T_{\mathrm{act}}=-|P\theta_{\Delta }+D\omega_{\Delta }|\right)$ or forward torque $\left(T_{\mathrm{act}}=|P\theta_{\Delta }+D\omega_{\Delta }|\right)$. A zero torque condition $\left(T_{\mathrm{act}}=0\right)$ corresponds to the periods when the active feedback controller is switched off in the intermittent control model (Asai et al., 2009). Action selection among these three choices is determined by the CBGT model as described below. For all simulations, the equation of motion and the equations governing spiking neural dynamics were discretized with a time step of δt=0.001s and integrated numerically using the forward Euler–Maruyama method.

### CBGT circuit model

#### Network architecture

**Figure 1.**
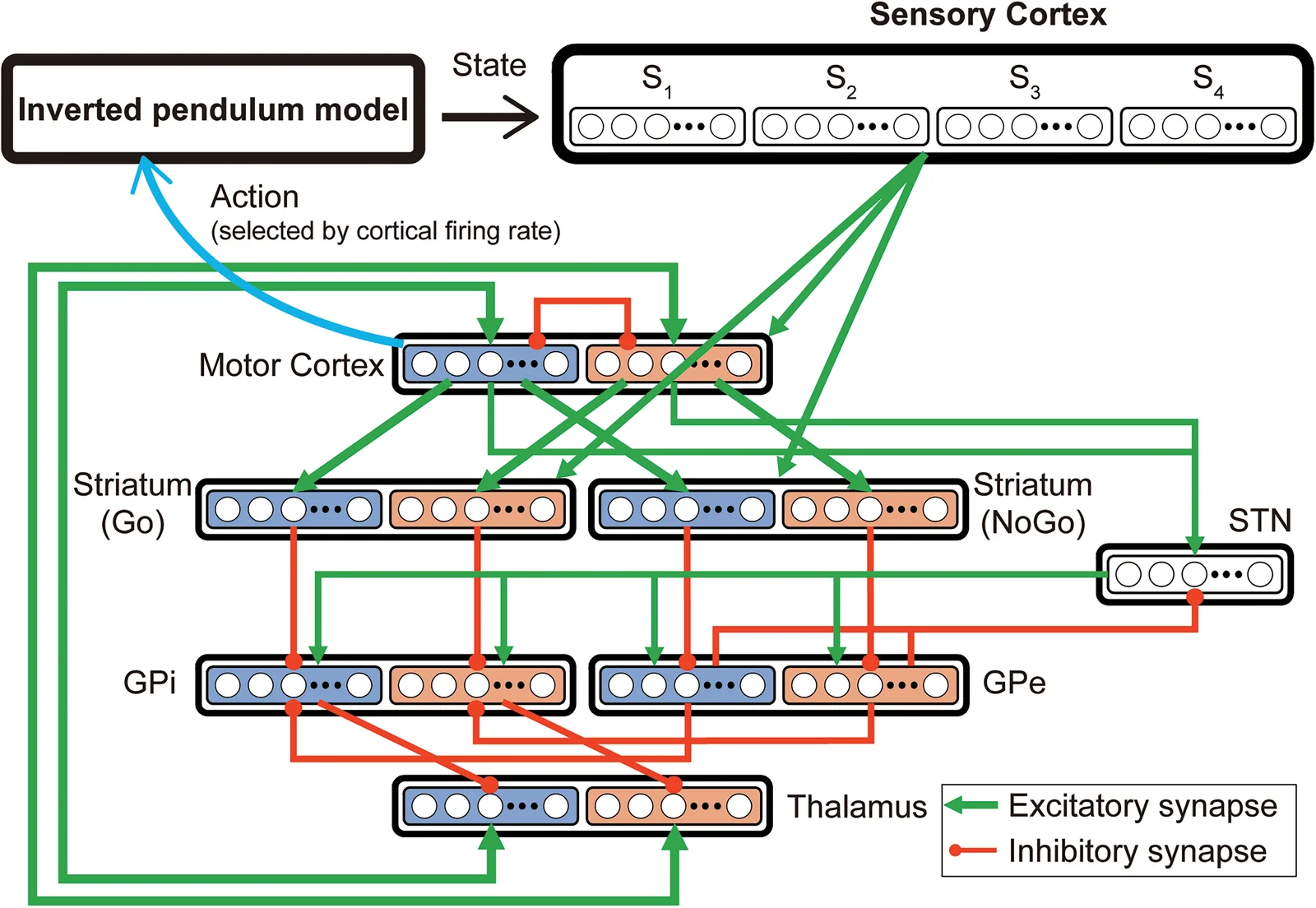
Schematic diagram of the network model for the cortico-basal ganglia-thalamic (CBGT) loop developed in this study. This model consists of neuronal populations of sensory cortex, cortex, striatum, globus pallidus pars externa (GPe), globus pallidus pars interna (GPi), subthalamic nucleus (STN), and thalamus. Each neural population except STN population includes two populations (colored blue and orange). Blue and orange populations are associated with backward- and forward-torque actions, respectively. Green lines with arrowheads represent excitatory injection and red lines with circular-heads inhibitory projections, respectively. Specifically, reciprocal excitatory connections exist between the motor cortex and thalamus, where thalamic neurons project back to their corresponding agonistic motor cortical subpopulations (e.g., backward thalamus to backward motor cortex) to complete the thalamocortical feedback loop. In this model, the state of the inverted pendulum is transmitted to and represented in the sensory cortex, and active torque (either backward torque, forward torque, or null torque) is selected by the spike rate of two populations of the cortical neurons.

The CBGT circuit model was structured as illustrated in Figure 1, following the topological framework of Ursino et al. (2020). The network processes the delayed physical state of the inverted pendulum and selects one of the three active torques $\left(T_{\mathrm{act}}\right)$ to maintain upright stability. Concurrently, the spiking dynamics of cortical populations generate local field potentials (LFPs) to simulate cortical EEG.

The model encompasses several interconnected neuronal populations: the sensory cortex, motor cortex, striatum, globus pallidus pars externa (GPe), globus pallidus pars interna (GPi), STN, and thalamus. The striatum is segregated into D1 receptor-expressing (Go-striatum, direct pathway) and D2 receptor-expressing (NoGo-striatum, indirect pathway) populations. Except for the STN, which comprises a single population of 500 neurons, each nucleus consists of 1,000 neurons split into two functionally distinct subpopulations of 500 neurons each: a “backward” population (Fig. 1, blue) and a “forward” population (Fig. 1, orange), corresponding to the actions for backward and forward torques, respectively. Neurons within the same subpopulation lack intra-group connectivity but project to their target agonistic subpopulations in an all-to-all manner. For instance, cortical neurons in the backward population project exclusively to backward-related subpopulations in both the Go- and NoGo-striatum. Excitatory and inhibitory pathways are modeled via glutamatergic and GABAergic projections, respectively (Fig. 1). Specifically, reciprocal excitatory connections exist between the motor cortex and thalamus, where thalamic neurons project back to their corresponding agonistic motor cortical subpopulations (e.g., backward thalamus to backward motor cortex) to complete the thalamocortical feedback loop. The sensory cortex contains 2,000 neurons, whose firing rates encode the delayed state variables ( $\theta_{\Delta }$ and $\omega_{\Delta }$) and drive downstream cortical and striatal populations.

In this modeling of embodied posture control, the spiking CBGT network incorporated both established neuroanatomy and functionally principled computational abstractions. In accordance with the known parallel-channel organization of the cortico-basal ganglia loop (Alexander and DeLong, 1985; Hoover and Strick, 1993), neurons in each CBGT subregion (motor and sensory cortices, striatum, GPe, STN, GPi, thalamus) are segregated into two distinct, nonoverlapping populations governing opposing movement directions: forward-torque channel and backward-torque channel. This dual-channel design provides a functional representation of the antagonist muscle pair acting at the ankle joint during quiet standing—namely, plantarflexors (soleus and gastrocnemius) resisting forward sway and dorsiflexors (tibialis anterior, TA) resisting backward sway. The anatomical separation of antagonist muscle representations in the primary motor cortex (Rathelot and Strick, 2006) and the ability of striatal microstimulation to elicit single-joint directional movements (Alexander and DeLong, 1985; Nambu et al., 2002) provide neurophysiological motivation for this channel segregation. For sensory state encoding, a mapping of a biomechanical state onto firing of spatially distributed neurons of sensory cortex serves as a functional abstraction representing multi-sensory integration (somatosensory, vestibular, and visual) conveyed to the striatum via sensory cortical inputs. Rather than modeling the full sensory cortical population, state-dependent corticostriatal projection weights W represent the functional tuning of sensory-to-striatal synaptic strength, encoding contextual state-action mappings across Go (Direct) and NoGo (Indirect) striatal spiny projection neurons.

#### Neuron model

Individual neurons are modeled as leaky integrate-and-fire (LIF) units without detailed biophysical ion-channel dynamics. The membrane potential integrates synaptic currents over time and fires an action potential upon reaching a fixed threshold. The LIF model accounts for a leak current that drives the potential toward its resting state (Izhikevich, 2004). The membrane potential V of a single neuron, as the time-dependent dynamic variable, evolves as follows (Ouyang et al., 2022):

$$\begin{array}{c} \tau \frac{dV}{dt}=-(V-V_{\mathrm{rest}})-g_{\mathrm{ex}}\;(V-E_{\mathrm{ex}})-g_{\mathrm{inh}}\;(V-E_{\mathrm{inh}})+I_{\mathrm{app}} \end{array},$$

where τ=20ms is the membrane time constant, $V_{\mathrm{rest}}=-60\;\mathrm{mV}$ is the resting potential, $E_{\mathrm{ex}}=0\;\mathrm{mV}$ is the excitatory reversal potential, and $E_{\mathrm{inh}}=-80\;\mathrm{mV}$ is the inhibitory reversal potential. The term $I_{\mathrm{app}}$ represents an external input current composed of a population-specific baseline constant (Table 1) and additive, independent Gaussian white noise. These population-specific constants reflect physiological background drives; for example, striatum neurons receive the dopaminergic bias (*D* = 1.0), while GPe and GPi neurons receive constant current to sustain spontaneous baseline firing.

**Table 1.** Parameters of the external input current $I_{\mathrm{app}}$, in a LIF neuron model

Neuron population	Value of the constant term
Go	3.25
NoGo	−3.9
GPe	18
GPi	24

Striatum (Go and NoGo) neurons receive dopaminergic input ( $I_{\mathrm{app}}=\alpha \cdot D$ with α=3.25 for Go neurons and $I_{\mathrm{app}}=\beta \cdot D$ with β=−3.9 for NoGo neurons, D = 1.0 in this study). Unlike the Ursino model, external inputs to Go neurons were assumed to be always excitatory for simplicity. NoGo neurons receive inhibitory input. Thus, the constant term (external input current $I_{\mathrm{app}}$) is always positive in Go neurons and negative for NoGo neurons. GPe (globus pallidus pars externa) and GPi (globus pallidus pars interna) receive excitatory background input to exhibit certain basal activity.

The time-dependent excitatory synaptic conductance $\left(g_{\mathrm{ex}}\right)$ for a given postsynaptic neuron is defined as follows:

$$\begin{array}{c} g_{\mathrm{ex}}(t)=\underset{i\in \mathrm{input}\;\mathrm{spikes}}{\sum }\;w_{i}u(t-t_{i})e^{-((t-t_{i})/\tau_{\mathrm{ex}})} \end{array},$$

where u(t) is the Heaviside step function ( u(t)=1 for t≥0, and 0 otherwise). The coefficient $w_{i}$ defines the synaptic coupling strength from the presynaptic neuron delivering the *i*-th spike at time $t_{i}$. These coupling weights depend on the identity of the presynaptic and postsynaptic populations (Tables 2–4). To introduce structural heterogeneity, the 500 × 500 connectivity matrix between populations was generated by scaling random numbers drawn from a Cauchy distribution by a population-specific coefficient (Tables 2–4). The excitatory decay constant was set to $\tau_{\mathrm{ex}}=5\;\mathrm{ms}$. The time-dependent inhibitory synaptic conductance $\left(g_{\mathrm{inh}}\right)$ was modeled analogously, with a decay constant of $\tau_{\mathrm{inh}}=10\;\mathrm{ms}$.

**Table 2.** Parameters of the synaptic weights in the present network model, which are common for the intermittent and continuous CBGT controller

Presynaptic neuron	Postsynaptic neuron	Synapse type	Coefficient name and value
Sensory cortex	Cortex	Excitatory	*W*_S2C_ 7.5 × 10^−3^
Cortex	Cortex	Inhibitory	*W*_LAT_ 7.5 × 10^−2^
Cortex	Go	Excitatory	*W*_C2G_ 2.54 × 10^−3^
Cortex	NoGo	Excitatory	*W*_C2N_ 4.475 × 10^−3^
Cortex	STN	Excitatory	*W*_C2STN_ 3.00 × 10^−2^
Cortex	Thalamus	Excitatory	*W*_C2T_ 4.655 × 10^−2^
Go	GPi	Inhibitory	*W*_G2I_ 4.35 × 10^−2^
NoGo	GPe	Inhibitory	*W*_N2E_ 2.6 × 10^−2^
GPe	GPi	Inhibitory	*W*_E2I_ 3.21 × 10^−2^
GPe	STN	Inhibitory	*W*_E2STN_ 2.085 × 10^−2^
GPi	Thalamus	Inhibitory	*W*_I2T_ 2.875 × 10^−2^
STN	GPe	Excitatory	*W*_STN2E_ 2.85 × 10^−2^
STN	GPi	Excitatory	*W*_STN2I_ 5.345 × 10^−2^
Thalamus	Cortex	Excitatory	*W*_T2C_ 9.64 × 10^−3^

The synaptic weight index of size 500 × 500 is generated by multiplying a random number that follows a Cauchy distribution by the coefficient values in this table.

**Table 3.** Parameters of the synaptic weight from the sensory cortex to the striatum in the intermittent CBGT model

State	Neuronal population	Action-related population	Coefficient name and value
*S*_1_	Go	Forward torque	*G*_min_ 3.0 × 10^−3^
		Backward torque	*G*_max_ 1.0 × 10^−2^
	NoGo	Forward torque	*N*_max_ 1.7 × 10^−2^
		Backward torque	*N*_min_ 5.0 × 10^−3^
*S*_2_	Go	Forward torque	*W*_S2G_ 6.5 × 10^−3^
		Backward torque	*W*_S2G_ 6.5 × 10^−3^
	NoGo	Forward torque	*W*_S2N_ 1.1 × 10^−2^
		Backward torque	*W*_S2N_ 1.1 × 10^−2^
*S*_3_	Go	Forward torque	*G*_max_ 1.0 × 10^−2^
		Backward torque	*G*_min_ 3.0 × 10^−3^
	NoGo	Forward torque	*N*_min_ 5.0 × 10^−3^
		Backward torque	*N*_max_ 1.7 × 10^−2^
*S*_4_	Go	Forward torque	*W*_S2G_ 6.5 × 10^−3^
		Backward torque	*W*_S2G_ 6.5 × 10^−3^
	NoGo	Forward torque	*W*_S2N_ 1.1 × 10^−2^
		Backward torque	*W*_S2N_ 1.1 × 10^−2^

**Table 4.** Parameters of the synaptic weight from the sensory cortex to the striatum in the forced-choice CBGT model

State	Neuronal population	Action-related population	Coefficient name and value
*S*_1_	Go	Forward torque	*G*_min_ 3.0 × 10^−3^
		Backward torque	*G*_max_ 1.0 × 10^−2^
	NoGo	Forward torque	*N*_max_ 1.7 × 10^−2^
		Backward torque	*N*_min_ 5.0 × 10^−3^
*S*_2_	Go	Forward torque	*G*_max_ 1.0 × 10^−2^
		Backward torque	*G*_min_ 3.0 × 10^−3^
	NoGo	Forward torque	*N*_min_ 5.0 × 10^−3^
		Backward torque	*N*_max_ 1.7 × 10^−2^
*S*_3_	Go	Forward torque	*G*_max_ 1.0 × 10^−2^
		Backward torque	*G*_min_ 3.0 × 10^−3^
	NoGo	Forward torque	*N*_min_ 5.0 × 10^−3^
		Backward torque	*N*_max_ 1.7 × 10^−2^
*S*_4_	Go	Forward torque	*G*_min_ 3.0 × 10^−3^
		Backward torque	*G*_max_ 1.0 × 10^−2^
	NoGo	Forward torque	*N*_max_ 1.0 × 10^−2^
		Backward torque	*N*_min_ 5.0 × 10^−3^

#### Physical state representation in the sensory cortex

**Figure 2.**
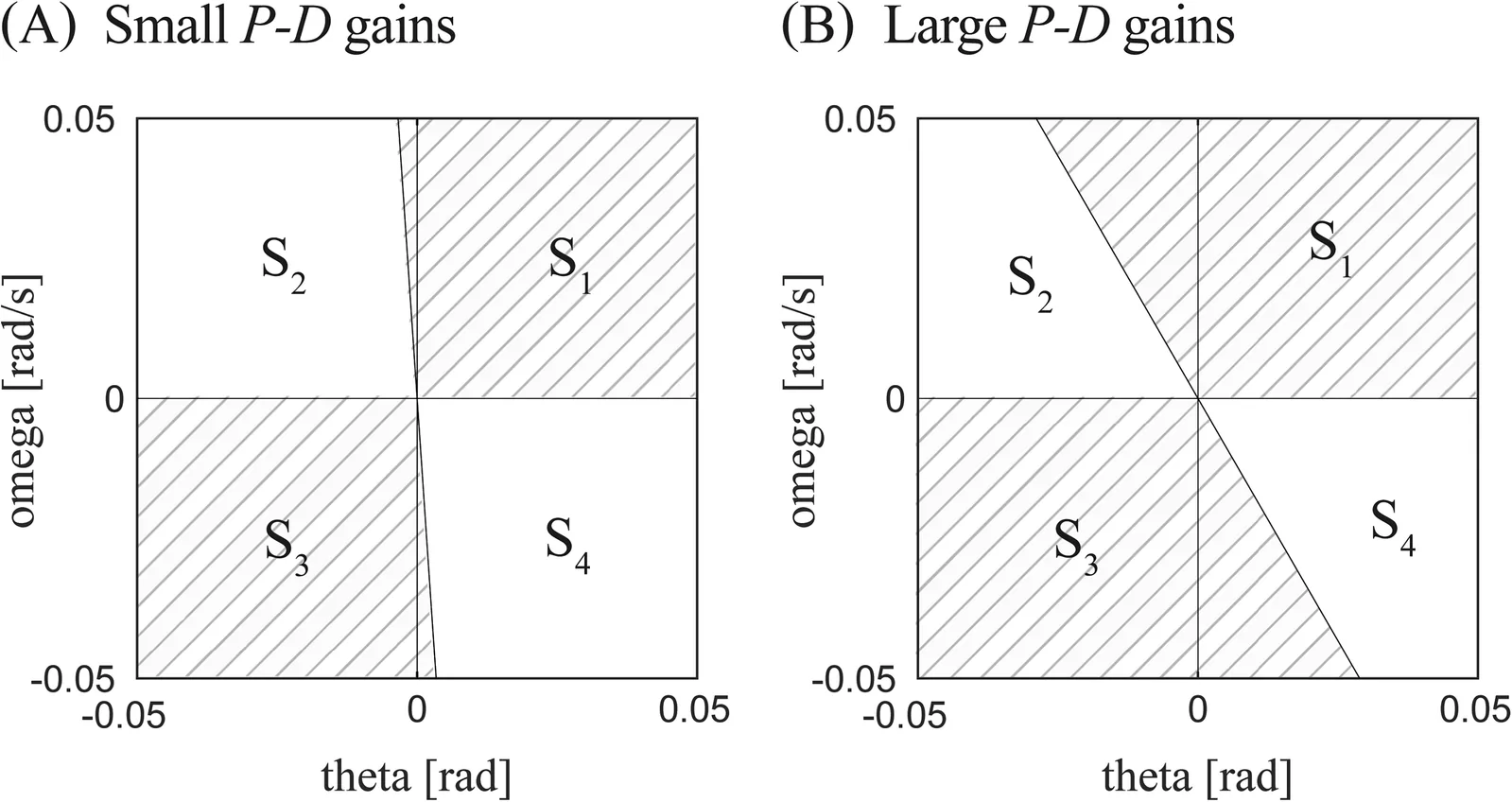
Categorical representation and encoding of the physical state of the inverted pendulum based on the position of the state point in the phase plane. The state of the inverted pendulum is encoded based on the four regions $\left(S_{1},S_{2},S_{3},S_{4}\right)$ in the phase plane. The negatively tilted line through the origin is defined as ω=−(P/D)θ, where (P,D)=(0.45mgh,18) in ***A*** with the small *P*-*D* gains and (P,D)=(0.5mgh,150) in ***B*** with the large *P*-*D* gains.

For computational simplicity, the delayed physical state of the pendulum $\left(\theta_{\Delta },\;\omega_{\Delta }\right)$ is categorized into four state-dependent regions (*S*_1_–*S*_4_) within the phase plane, corresponding roughly to the four quadrants (Fig. 2). Specifically, the phase space is partitioned by the lines $\omega_{\Delta }=-(P/D)\theta_{\Delta }$ and the *θ*-axis into the following four regions:

*S*_1_: $\omega_{\Delta }>-(P/D)\theta_{\Delta }$ and $\theta_{\Delta }>0$
*S*_2_: $\omega_{\Delta }\leq -(P/D)\theta_{\Delta }$ and $\theta_{\Delta }\geq 0$(4)
*S*_3_: $\omega_{\Delta }\leq -(P/D)\theta_{\Delta }$ and $\theta_{\Delta }<0$
*S*_4_: $\omega_{\Delta }>-(P/D)\theta_{\Delta }$ and $\theta_{\Delta }<0$

The 2,000 sensory cortical neurons are divided into four identical pools of 500 neurons, each dedicated to one of these regions. When the delayed state resides within region *S_i_* (*i* = 1,2,3,4), the corresponding pool of 500 neurons fires stochastically according to an independent Poisson process, thereby mapping the system state to sensory firing rates. The probability of a single neuron generating a spike within a time step δt=0.001s is given by the following (Johnson, 1996):

$$\begin{array}{c} \mathrm{Prob}[N_{\mathrm{spike}}(t+\delta t)-N_{\mathrm{spike}}(t)=1]=\lambda \delta t, \end{array}$$

where $N_{\mathrm{spike}}(t)$ is the cumulative number of firings for a neuron until time t and λ is the constant Poisson intensity (firing rate). We set λ=100Hz for neurons assigned to the active region *S_i_* and λ=0Hz otherwise. In Experiment 2, we also tested λ=200Hz to assess how sensory signal intensity affects the simulated EEG.

### Implementation of the intermittent and forced-choice continuous-like CBGT controllers

We configured the network parameters to realize two different control regimes for postural stabilization: an intermittent controller and a forced-choice continuous-like controller. These regimes were implemented by systematically altering the synaptic weights W for the sensorimotor corticostriatal projections. As mentioned above, we employed two sets of typical *P*-*D* feedback gains and noise intensity (σ): a small gains-noise set and a large gain-noise set, respectively, based on the previous study that assimilates postural sway data from healthy people and patients with Parkinson's disease with severe postural impairment (Suzuki et al., 2020).

#### Intermittent CBGT controller

In the intermittent control framework, the active feedback controller is switched off when the state point of the pendulum moves near the stable manifold of the unstable, saddle-type upright equilibrium (Asai et al., 2009). Regions *S*_2_ and *S*_4_ represent these “safe” zones, where the passive dynamics transiently pull the pendulum back toward the vertical position without active intervention. Conversely, the active feedback controller is switched on when the state point of the pendulum enters regions *S*_1_ or *S*_3_, which lie near the unstable manifold.

The motor cortex evaluates and selects the appropriate motor command through firing rate competition between spike rates of the backward and forward populations. Sensory signals generated in the sensory cortex drive this loop by stimulating the corresponding Go or NoGo striatal subpopulations. These signals propagate through the GPe, GPi, and STN to the thalamus, ultimately feeding back to the cortex via thalamocortical projections. Excitatory PSCs from these projections accumulate in the cortical neurons. Firing rate competition operates between the aggregate spike counts per second of the backward and forward cortical populations. To ensure high temporal resolution in action selection, firing rate and motor commands are evaluated and updated continuously at every simulation time step (δt=0.1ms). That is, at each time step t, the population firing rate of the motor cortex is computed using a 0.1-s moving time window ([t−0.1s,t]). If this smoothed firing rate exceeds the decision threshold, an active feedback torque is exerted on the ankle joint. Thus, the temporal resolution of the action-selection process matches the numerical integration step, ensuring that the observed intermittency in torque generation arises dynamically from the network's spiking threshold dynamics rather than from discrete temporal sampling.

More specifically, in the context of a drift-diffusion model, an increase in a population's firing rate represents the accumulation of evidence toward its corresponding action. If the firing rate of the backward population reaches the decision threshold (50 Hz) first, the backward torque $\left(T_{\mathrm{act}}=-|P\theta_{\Delta }+D\omega_{\Delta }|\right)$ is applied to the pendulum. Conversely, if the forward population reaches the threshold first, the forward torque $\left(T_{\mathrm{act}}=|P\theta_{\Delta }+D\omega_{\Delta }|\right)$ is executed. In this study, the threshold for active torque selection was set at 50 Hz throughout the study. The threshold was fixed at 50 Hz based on established studies (Mohanty et al., 2025). The selected torque remains active as long as the winning population's firing rate stays above the threshold. A period during which both populations remain below the 50 Hz threshold defines the DT, during which no active torque is applied $\left(T_{\mathrm{act}}=0\right)$, implementing the “control-off” phase of the intermittent strategy. Reciprocal inhibition between the two cortical populations enforces a winner-take-all dynamic (Ursino et al., 2020; Fig. 1).

**Figure 3.**
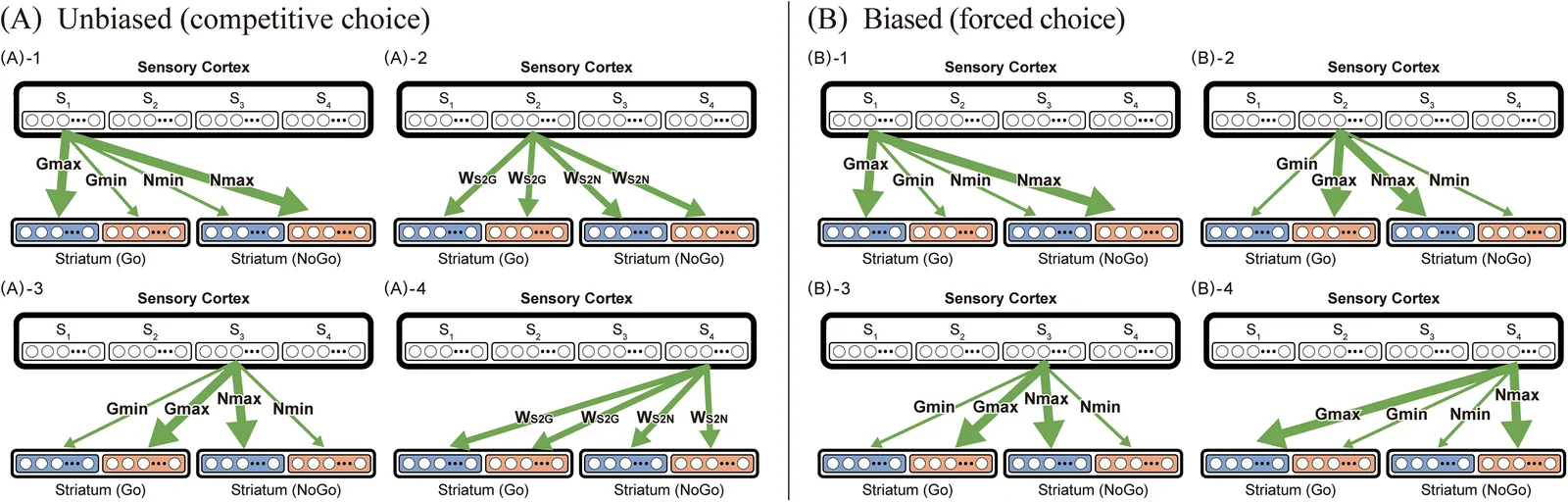
The state-dependent synaptic weights of the sensory corticostriatal projections for the intermittent and forced-choice control model. ***A***, The intermittent control model. ***B***, The forced-choice continuous-like control model. ***A***, Characteristics of the unbiased synaptic weight vector $W_{\mathrm{unbiased}}$: When the pendulum is in *S*_1_ as ***A-1***, the backward and forward population of Go-striatum receives strong and weak inputs, respectively, and the backward and forward population of NoGo-striatum receives weak and strong inputs, respectively. When the pendulum is in *S*_3_, as ***A-3***, the forward population of the Go-striatum and the backward population of the NoGo-striatum receive strong inputs, and the backward population of the Go-striatum and the forward population of the NoGo-striatum receive weak inputs. When the pendulum is in *S*_2_ or *S*_4_ as ***A-2*** and ***A-4***, the sensory corticostriatal inputs to the antagonistic populations were set with equal strength. ***B***, Characteristics of the biased synaptic weight vector $W_{\mathrm{biased}}$: When the pendulum is in *S*_1_ or in *S*_4_ as ***B-1*** and ***B-4***, the backward and forward population of the Go-striatum receives strong and weak inputs, respectively, and the backward and forward population of the NoGo-striatum receives weak and strong inputs, respectively. When the pendulum is in *S*_2_ or *S*_3_, as ***B-2*** and ***B-3***, the forward population of the Go-striatum and the backward population of the NoGo-striatum receive strong inputs, and the backward population of the Go-striatum and the forward population of the NoGo-striatum receive weak inputs.

**Figure 4.**
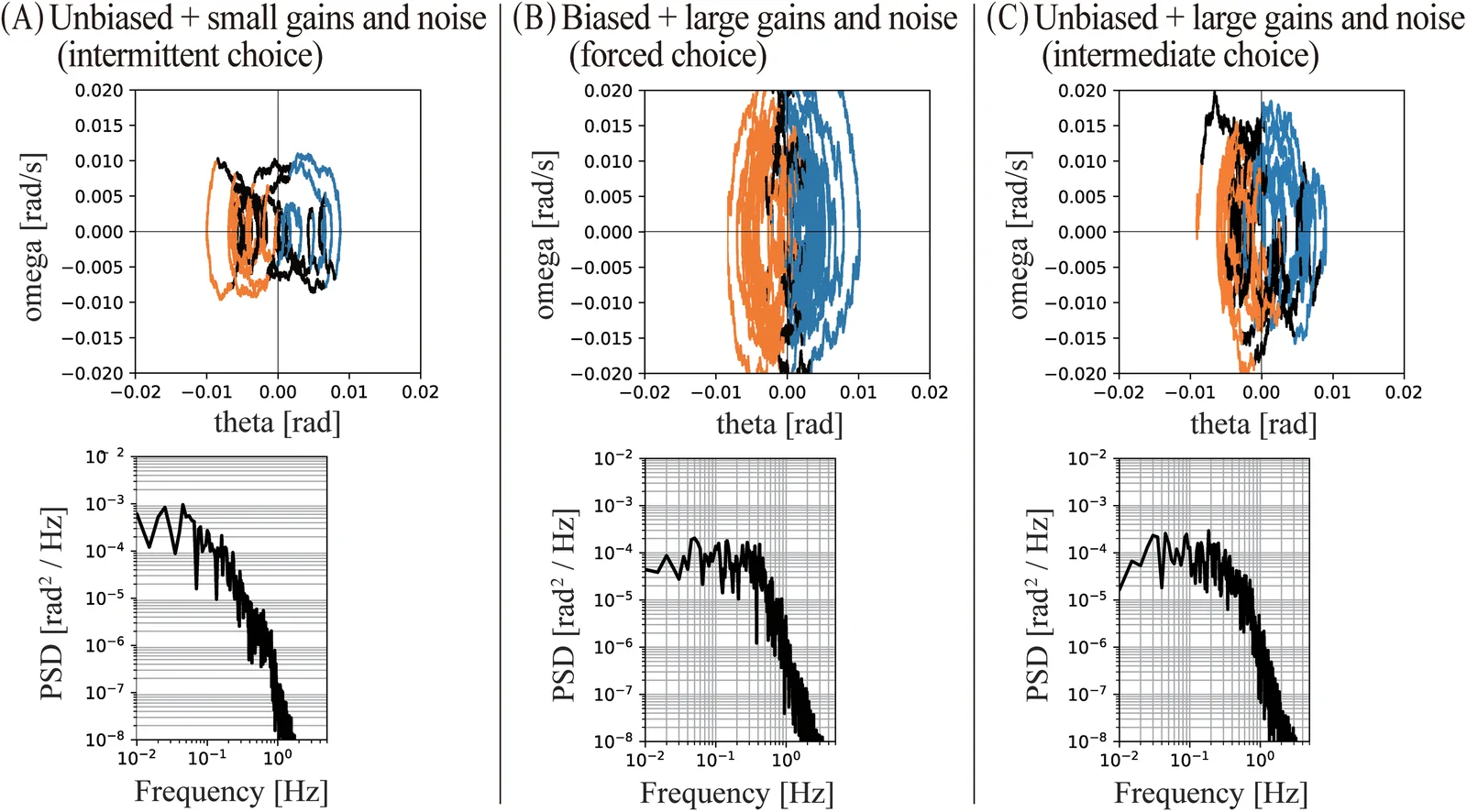
Dynamics of the inverted pendulum controlled by variants of the CBGT controllers. ***A***, The intermittent control model implemented with unbiased weight vector $W_{\mathrm{unbiased}}$ and small feedback gains and noise $\left(P_{\mathrm{small}},D_{\mathrm{small}},\sigma_{\mathrm{small}}\right)$. ***B***, The forced-choice continuous-like control model implemented with biased weight vector $W_{\mathrm{biased}}$ and large feedback gains and noise $\left(P_{\mathrm{large}},D_{\mathrm{large}},\sigma_{\mathrm{large}}\right)$. ***C***, A type of intermediate-choice model with unbiased weight vector $W_{\mathrm{unbiased}}$ and large feedback gains and noise $\left(P_{\mathrm{large}},D_{\mathrm{large}},\sigma_{\mathrm{large}}\right)$. The top panels are the phase portrait with a simulated trajectory for 40 s. Blue, orange, and black segments represent dynamics of the pendulum controlled by the backward-torque action, the forward-torque action, and the null action of the CBGT controller, respectively. Bottom panels are ensemble-averaged PSD of the tilt angle time series for 200 s across 30 simulation trials.

Because sensory corticostriatal weights W govern the drift bias in this competition, we explored these connections to satisfy the intermittent control criteria. More specifically, we set the small *P*-*D* gains (and noise intensity) in the model, by which upright posture of the inverted pendulum cannot be stabilized, if the small-gain delay PD feedback controller operates persistently, due to delay-induced instability. Thus, the exploration was performed to find W that could stabilize the upright posture, not to reproduce beta modulations in the simulated EEG, which was theoretically equivalent to finding W that could realize intermittent control. In this way, the appropriate weights W realize the following characteristics. When the pendulum enters *S*_1_ (tilted forward and falling), the backward and forward population of Go-striatum receives strong and weak inputs, respectively, ensuring a high probability of selecting backward torque. Concurrently, the backward and forward populations of the NoGo-striatum receive weak and strong inputs, respectively, preventing the antagonistic action from interfering with the command (Fig. 3*A-1*, Table 3). Conversely, when the pendulum is in *S*_3_ (tilted backward and falling), the forward Go-striatum and the backward NoGo-striatum receive strong inputs, while their counterparts receive weak inputs, favoring forward torque (Fig. 3*A*-*3*, Table 3). Crucially, when the trajectory enters the safe regions (*S*_2_ or *S*_4_), sensory inputs to the antagonistic striatal populations are balanced with equal strength. This unbiased projection ensures close, prolonged competition between the populations (Figs. 3*A-2*, *A-4*; Table 3), extending the DT and maximizing the probability of selecting the null action $\left(T_{\mathrm{act}}=0\right)$. The sensoristriatal synaptic weight vector W that satisfied the characteristics detailed here is referred to as $W_{\mathrm{unbiased}}$, which leads to balanced, unbiased action selections.

The exploration of the sensoristriatal synaptic weight vector $W_{\mathrm{unbiased}}$ to implement the intermittent control model was performed under the small fixed feedback parameters to (P,D)=(0.45mgh,18), with which the upright equilibrium remains unstable (unstable focus) even if the underlying PD controller operates continuously. Moreover, the noise amplitude was set to σ=0.2Nm. These values were consistent with the feedback gains and noise intensity identified by the data assimilation of postural sway data from healthy people (Suzuki et al., 2020).

#### Forced-choice (continuous-like) controller–baseline regime

To examine how sway-related beta modulation depends on the temporal structure of action selection within the model, we compared the balanced intermittent control regime $\left(W_{\mathrm{unbiased}}\right)$ against a sustained active control condition, termed the forced-choice (continuous-like) regime, in which the sensoristriatal synapse leads to biased action selections by the use of a biased synaptic weight vector, referred to as $W_{\mathrm{biased}}$.

It is important to clarify that this forced-choice regime is implemented within the identical spiking CBGT network structure by biasing the corticostriatal weight matrix $\left(W_{\mathrm{biased}}\right)$ toward the Direct (Go) pathway. This configuration forces the cortical population to persistently cross the firing rate threshold and generate active torque whenever the body deviates from equilibrium, thereby eliminating the prolonged Go/NoGo competitive dynamics (eliminating or narrowing DT) seen in safe sway regions $\left(S_{2},S_{4}\right)$. This regime serves as an internal computational baseline to evaluate how LFP spectral properties change when subcortical DT is minimized, rather than representing a literal implementation or critique of classical continuous feedback control models (Peterka, 2002).

The forced-choice controller was implemented by exploring $W_{\mathrm{biased}}$ that enforces a biased choice across all regions of the phase plane, thereby minimizing the null-action periods. When the pendulum is in *S*_1_ (tilted forward, falling forward) or in *S*_4_ (tilted forward, moving backward), the network parameters favor backward torque by routing strong inputs to the backward Go-striatum and forward NoGo-striatum and weak inputs to their counterparts (Figs. 3*B-1*, *B-4*; Table 4). Conversely, in regions *S*_2_ (tilted backward, moving forward) and in *S*_3_ (tilted backward, falling backward), the projections favor forward torque (Figs. 3*B-2*, *B-3*; Table 4). Under this parameter configuration, cortical action selection resembles a forced-choice between opposing active torques. Consequently, the DT is markedly shortened, the probability of selecting the null action becomes negligible, and the system approximates continuous active control within the CBGT architecture.

To prevent delay-induced instability under the delayed continuous feedback configuration used here, the feedback gains must be substantially higher than those of the intermittent controller (Asai et al., 2009). We therefore used (P,D)=(0.5mgh,150), which yields a stable upright equilibrium under uninterrupted feedback. Moreover, the noise amplitude was increased to σ=0.6Nm. These values are in accordance with the previous continuous control simulations to reproduce postural sway in patients with Parkinson's disease (Suzuki et al., 2020).

### Physiological and computational rationales for system parameters

The key operational parameters of the CBGT closed-loop model were defined based on established neurophysiological and biomechanical empirical data:

1. Decision threshold for torque generation: Action selection was modeled using a firing rate accumulator threshold mechanism (Tanaka, 2007). Active torque generation was triggered when M1 population firing rates crossed a decision threshold. While set to 50 Hz in main simulations, sensitivity analyses confirmed that lowering the threshold to 40 Hz produced identical, qualitative sway-phase-locked beta-band ERD/ERS modulations, confirming that beta dynamics are robust and independent of specific threshold values.
2. Temporal smoothing and delays: Cortical output firing rates were calculated using a 0.1 s moving average window to model the postsynaptic integration kinetics of cortical and motoneuronal dendrites. A total loop latency of 200 ms was incorporated to represent the long-loop transcortical/CBGT feedback delay encompassing sensory processing, subcortical action selection, and neuromuscular lag (Loram et al., 2005b).
3. Biomechanical constraints: Passive ankle joint stiffness was set to 80% of gravitational overturning torque (mgh), reflecting empirical measurements in humans showing that passive tissue elasticity alone is insufficient for upright stability (Loram and Lakie, 2002; Casadio et al., 2005). Sway dynamics $\left(\theta ,\dot{\theta }\right)$ were evaluated relative to the upright equilibrium state (θ=0) (Peterka, 2002). Sensitivity analyses confirmed that that varying passive stiffness within the physiologically reported range, 80–90% of mgh (Loram and Lakie, 2002), produced consistent postural stabilization and robust, identical sway-phase-locked beta-ERD/ERS dynamics.

### Simulation protocols and data analysis

#### Simulation parameters and testing trials

We defined a localized rectangular boundary in the phase plane ( 0≤|θ|≤0.05rad and 0≤|ω|≤0.05rad/s) centered at the origin; trajectories escaping this domain were classified as falls (postural failure). Each trial was initiated from a random position within a narrow region ( 0≤|θ|≤0.02rad and 0≤|ω|≤0.02rad/s). A simulation trial was considered successful if the pendulum avoided falling for 205 s, and this protocol was repeated to gather 30 successful trials. Data from the first 5 s of each trial were excluded from analysis to eliminate transient initialization artifacts.

#### Simulated EEG reconstruction

To assess beta-band desynchronization and synchronization, we simulated cortical EEG using the LFP generated by the motor cortex. The LFP signal was computed by summing all excitatory and inhibitory synaptic currents received across the entire cortical population (Ouyang et al., 2022):

$$\begin{array}{c} \mathrm{LFP}=\frac{1}{N}\overset{N}{\underset{i}{\sum }}\;\{\left|g_{i,\mathrm{ex}}\;(E_{\mathrm{ex}}-V_{i})|+|g_{i,\mathrm{inh}}\;(E_{\mathrm{inh}}-V_{i})\right|\} \end{array},$$

where $g_{i,\mathrm{ex}}$, $g_{i,\mathrm{inh}}$, and $V_{i}$ represent the time-dependent excitatory synapse conductance, the time-dependent inhibitory synapse conductance, and the membrane potential (served as a dynamic variable) of *i*th cortical neuron, respectively. ( $E_{\mathrm{ex}}$ and $E_{\mathrm{inh}}$ are constants as defined above.) Neurons from both the backward and forward cortical subpopulations were pooled indiscriminately, yielding a total population size of N=1,000.

### Sensitivity analysis of corticostriatal weights and decoupling from mechanical gains

To evaluate the sensitivity and robustness of the intermittent control regime and sway-related beta modulation to corticostriatal projection strengths, we performed a systematic parameter sweep. The key functional difference between the unbiased condition $\left(W_{\mathrm{unbiased}}\right)$ and the biased/forced-choice condition $\left(W_{\mathrm{biased}}\right)$ lies in the eight-dimensional weight vector W governing sensory cortical inputs to striatal Direct (Go) and Indirect (NoGo) pathways across phase regions ( $S_{2}$ and $S_{4}$).

We defined a linear interpolation path in this 8D weight space parameterized by s∈[0,1]:

$$W(s)=(1-s)W_{\mathrm{unbiased}}+sW_{\mathrm{biased}},$$

where s=0 corresponds to $W_{\mathrm{unbiased}}$ and s=1 to $W_{\mathrm{biased}}$. We evaluated five evenly spaced intermediate weight vectors ( $W_{1}$ to $W_{5}$ corresponding to s=1/6,2/6,3/6,4/6,5/6, respectively).

To test whether beta modulation depends solely on synaptic weight design or on the closed-loop interaction between neural competition and physical body movement, we independently fully crossed these seven weight configurations $\left(W_{\mathrm{unbiased}},W_{1}-W_{5},W_{\mathrm{biased}}\right)$ with two distinct mechanical feedback parameter sets (P,D,σ):

1. Healthy/small feedback regime $\left(P_{\mathrm{small}},D_{\mathrm{small}},\sigma_{\mathrm{small}}\right)$: (P,D)=(0.45mgh,18), noise amplitude σ=0.2Nm/rad, derived from data assimilation of healthy human sway during quiet standing (Suzuki et al., 2020).
2. PD/large feedback regime $\left(P_{\mathrm{large}},D_{\mathrm{large}},\sigma_{\mathrm{large}}\right)$: (P,D)=(0.5mgh,150), noise amplitude σ=0.6Nm/rad, representing the hyper-stiffness and exaggerated noise observed in severe parkinsonian postural impairment (Suzuki et al., 2020).

### Evaluation of the CBGT circuit topology (Experiment 2)

In Experiment 1, the network architecture could not be altered because the CBGT controller was strictly constrained by the requirement to stabilize the pendulum. To systematically evaluate how specific loops within the CBGT circuit shape the simulated EEG, Experiment 2 introduced targeted structural dissections and parameter modifications. To maintain consistent sensory drive despite the loss of postural stability caused by these perturbations, the pendulum was driven passively along a predefined periodic trajectory, decoupled from the cortical motor command.

The sensory feedback pathway remained intact, allowing state-dependent signals from the sensory cortex to drive the network exactly as in Experiment 1. For conditions utilizing the intermittent CBGT parameters, i.e., with the unbiased synaptic weight vector $W_{\mathrm{unbiased}}$, i.e., Conditions 1 (the intermittent CBGT controller as is), 3 (firing rate of sensory cortex is altered to *λ* = 200), 4 (projections from the sensory cortex to the motor cortex and the striatum were dissected), 5 (projections from the motor cortex to STN were dissected), and 6 (the local feedback projections from GPe to STN were dissected), the passive trajectory alternated between regions *S*_1_ and *S*_4_ with a 5 s period (switching every 2.5 s), mimicking the characteristic phase-plane trajectories observed in Experiment 1 (Fig. 6*A*). For the forced-choice continuous-like control configuration, i.e., with the biased synaptic weight vector $W_{\mathrm{biased}}$, (Condition 2), the passive trajectory routed the system through *S*_1_ and *S*_4_ for 1.25 s each, followed by *S*_3_ for the remaining 2.5 s (total period of 5 s), approximating the elliptical trajectory in Figure 6*B*. Each simulation ran for 5,000 s to yield 1,000 complete cycles (epochs) for robust spectral analysis. We evaluated the following six distinct network conditions:

1. [Condition 1] Baseline intermittent CBGT controller with $W_{\mathrm{unbiased}}$ under passive periodic movement between *S*_1_ and *S*_4_ (period: 5 s).
2. [Condition 2] Baseline forced-choice continuous-like CBGT controller with $W_{\mathrm{biased}}$ under passive periodic movement through *S*_1_, *S*_4_, and *S*_3_ (period: 5 s).
3. [Condition 3] Same configuration as Condition 1 with $W_{\mathrm{unbiased}}$, but with the sensory cortex firing rate increased to *λ* = 200 Hz to analyze the impact of sensory signal intensity.
4. [Condition 4] Same configuration as Condition 1 with $W_{\mathrm{unbiased}}$, but with the thalamocortical projections disconnected to open the major cortico-basal ganglia feedback loop.
5. [Condition 5] Same configuration as Condition 1 with $W_{\mathrm{unbiased}}$, but with the pathway from the cortex to the STN disconnected, eliminating STN activity to simulate a network lacking the hyperdirect pathway.
6. [Condition 6] Same configuration as Condition 1 with $W_{\mathrm{unbiased}}$, but with the local inhibitory connections from the GPe and the STN disconnected (no GPe–STN loop).

### Signal processing and statistical averaging

Time series data—including pendulum kinematics, active torques, and cortical LFPs—were extracted over cumulative durations of 2,050 s for Experiment 1 and 5,050 s for Experiment 2 (combining 10 separate simulation runs per condition, discarding the initial 5 s transient phase).

For Experiment 1, individual fall-recovery cycles were isolated. Each cycle was defined as beginning at the onset of a micro-fall and terminating at the completion of the subsequent micro-recovery. To isolate prominent postural adjustments, the start and end points of these cycles were detected using the scipy.signal.find_peaks() function in Python, applied to the absolute state vector |θ(t)−ε| where *ε* = 0.001 rad is a small baseline offset used to focus trajectory-arcs locating slightly away from the upright position. We extracted cycles exhibiting a sway amplitude greater than 0.001 rad and a duration longer than 0.05 s to filter out minor chattering. For Experiment 2, epoch segmentation was defined directly by the fixed 5 s period of the passive driving trajectory.

Time–frequency representations of the LFP were computed using a short-time Fourier transform (STFT) with a 1 s sliding window. To analyze event-related spectral perturbations (ERSPs), the time-averaged power across the entire trial was established as the baseline for each frequency, and dynamic power fluctuations were converted to decibels (dB) relative to this baseline (Nakamura et al., 2023). Because individual fall-recovery cycles in Experiment 1 varied in duration, the kinematics, torque profiles, and ERSP matrices were time-warped. The micro-fall and micro-recovery intervals were normalized separately so that their lengths matched the respective mean ensemble durations across all extracted epochs before computing the final event-locked average. For Experiment 2, ERSPs and population-specific firing rates were directly ensemble-averaged over the fixed 1,000 epochs. All computational modeling and data analysis pipelines were executed using Python (version 3.10.13).

### Code accessibility

The custom computer code and algorithms generated during the current study are publicly available in the GitHub repository (https://github.com/nomura-taishin/cbgt-intermittent-postural-control/).

## Results

### Distinct postural stabilization and sway kinematics under intermittent and forced-choice continuous-like CBGT controllers

To evaluate the behavioral consequences of different neural control strategies, we first analyzed the phase-plane trajectories and power spectral densities (PSDs) of the inverted pendulum stabilized by the intermittent (with $W_{\mathrm{unbiased}}$) and forced-choice continuous-like (with $W_{\mathrm{biased}}$) CBGT controllers (Fig. 4). The intermittent control model exhibited a characteristic butterfly-wing-shaped trajectory in the phase portrait (Fig. 4*A*, top panel). This pattern indicates that the pendulum achieved stability by exploiting the stable manifold of the saddle-type upright equilibrium point, as evidenced by the hyperbolic arcs centered at the origin during the control-off (DT) periods (represented by black segments). Furthermore, the low-frequency band of the angle PSD for the intermittent model exhibited a 1/*f*-like power-law scaling (Fig. 4*A*, bottom panel), a well-established computational hallmark of intermittent control strategies (Asai et al., 2009; Nomura et al., 2022).

In contrast, the forced-choice continuous-like control model displayed an elliptical spiral trajectory (Fig. 4*B*, top panel). In this regime, the system dynamics were continuously dominated by alternating active commands—backward torque in the right half-plane and forward torque in the left half-plane—with negligible null-action intervals between them (Fig. 4*B*, top panel). Reflecting this uninterrupted feedback regulation, the low-frequency regime of the angle PSD exhibited a plateau characteristic of white noise (Fig. 4*B*, bottom panel). This profile resembles the low-frequency spectral flattening and more rigid sway patterns previously reported in patients with advanced Parkinson's disease (Suzuki et al., 2020; Nomura et al., 2022). Together, these behavioral simulations confirm that functionally tuning the corticostriatal synaptic weights is sufficient to switch the closed-loop system between intermittent and forced-choice continuous-like control regimes with distinct sway dynamics.

### Sway-coupled spiking modulations and phasic cortical LFPs emerged under intermittent control (Experiment 1)

**Figure 5.**
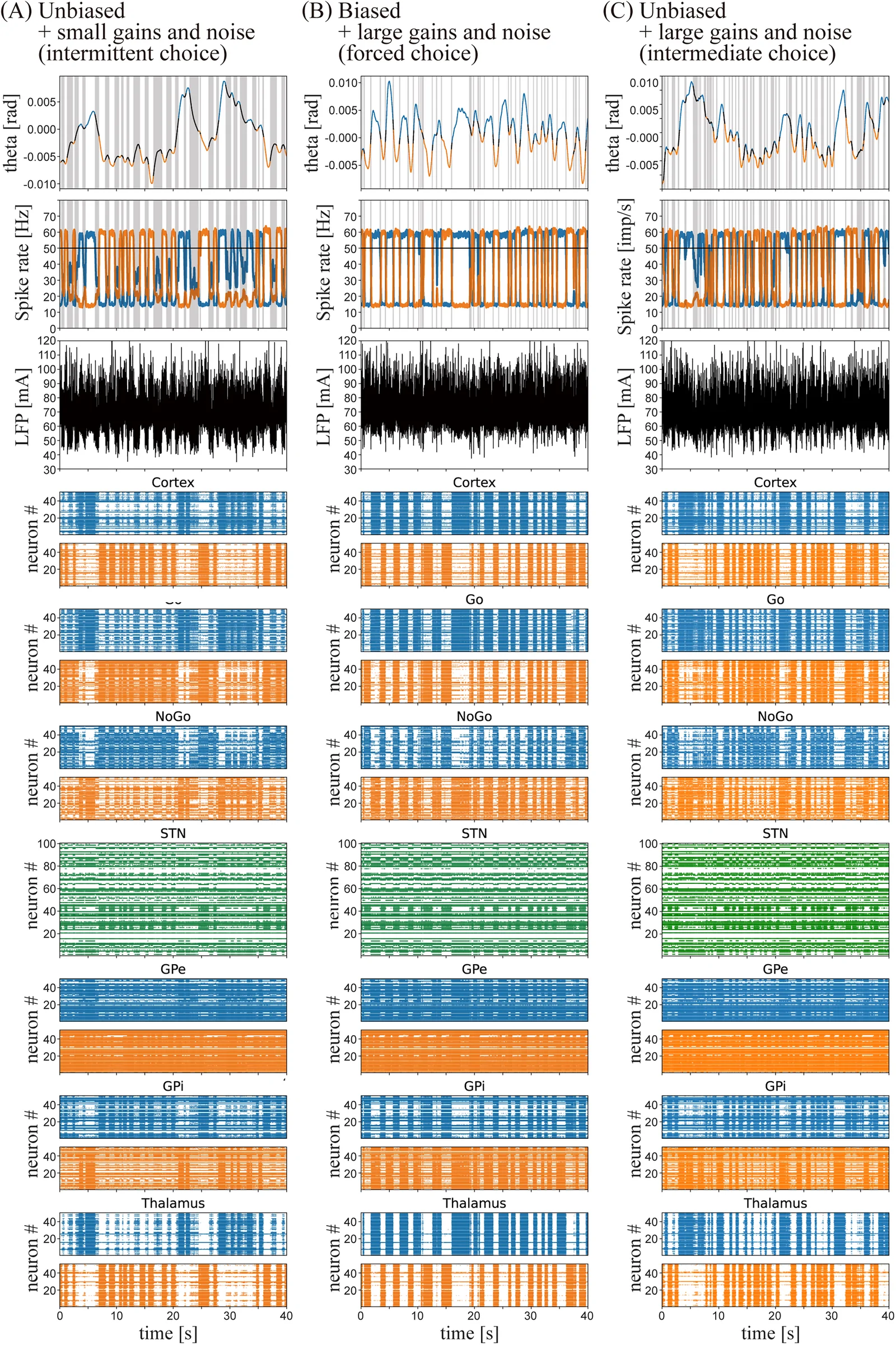
Model-simulated sway and internal neural dynamics over multiple fall-recovery cycles. ***A***, The intermittent control model implemented with $W_{\mathrm{unbiased}}$ and $\left(P_{\mathrm{small}},D_{\mathrm{small}},\sigma_{\mathrm{small}}\right)$. ***B***, The forced-choice continuous-like control model implemented with $W_{\mathrm{biased}}$ and $\left(P_{\mathrm{large}},D_{\mathrm{large}},\sigma_{\mathrm{large}}\right)$. ***C***, A type of intermediate-choice model with $W_{\mathrm{unbiased}}$ and $\left(P_{\mathrm{large}},D_{\mathrm{large}},\sigma_{\mathrm{large}}\right)$. Top trace, The time series of the tilt angle with the color codes representing the choice of actions as in Figure 4. Second trace, Spike rates of the backward and forward populations in the cortex. Gray-shaded vertical bars represent a sequence of decision times (DT). Third trace, LFP as the simulated EEG. Panels at the 4th to the 10th row (expect 7th). Raster plots of spikes for 50 neurons out of 500 neurons in each of the backward and forward populations for the cortex, Go-striatum, NoGo-striatum, STN, GPe, GPi, and thalamus. The 7th row is for 100 STN neurons out of 1,000 neurons.

**Figure 6.**
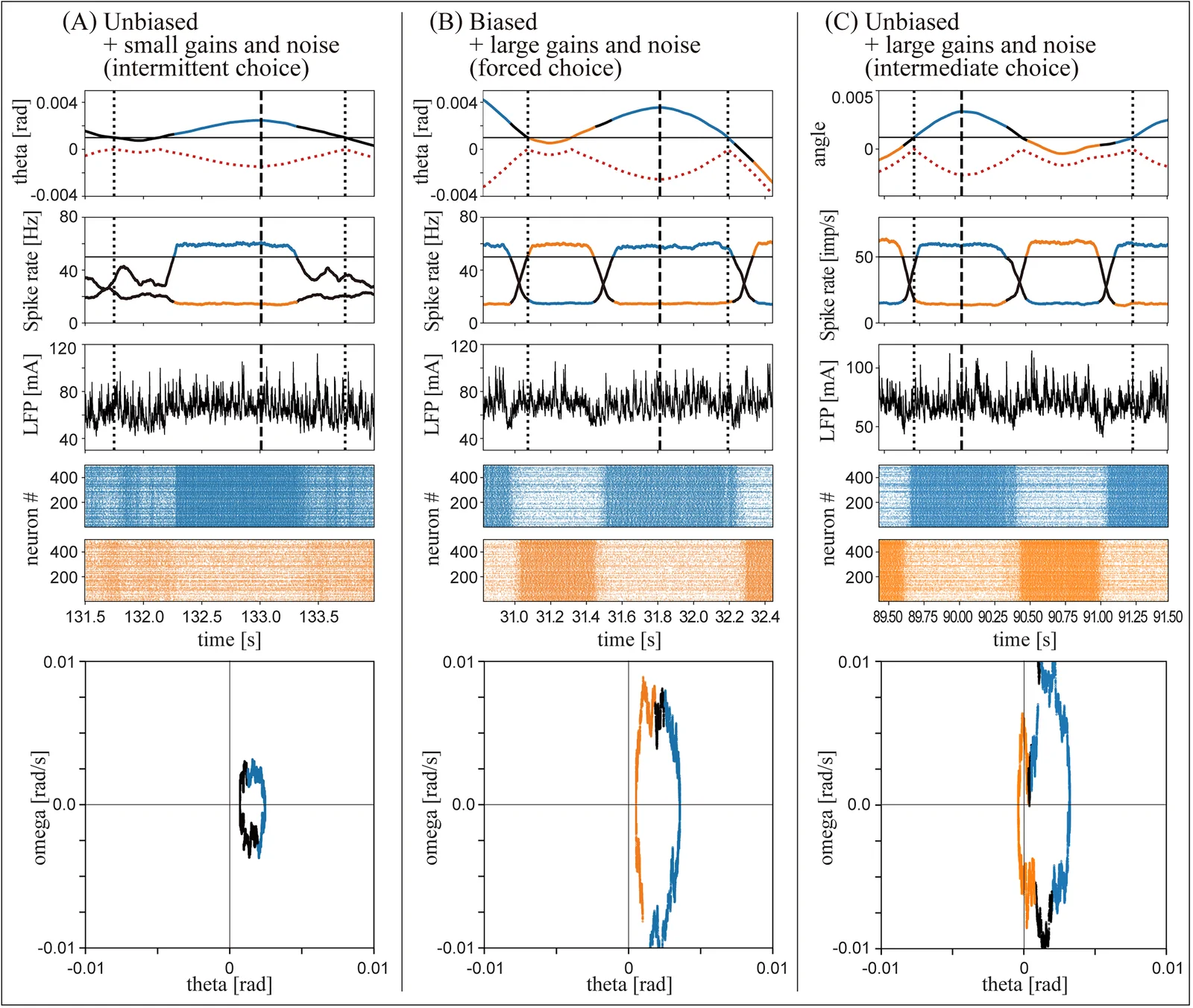
Model-simulated sway and neural dynamics for a single extracted fall-recovery cycle along with the accompanying changes in the spike rates. ***A***, The intermittent control model with $W_{\mathrm{unbiased}}$ and $\left(P_{\mathrm{small}},D_{\mathrm{small}},\sigma_{\mathrm{small}}\right)$. ***B***, The forced-choice continuous-like control model with $W_{\mathrm{biased}}$ and $\left(P_{\mathrm{large}},D_{\mathrm{large}},\sigma_{\mathrm{large}}\right)$. ***C***, A type of intermediate-choice model with $W_{\mathrm{unbiased}}$ and $\left(P_{\mathrm{large}},D_{\mathrm{large}},\sigma_{\mathrm{large}}\right)$. Spike rates of the backward and forward cortical populations (2nd row), and the corresponding LFP time series (3rd row) and raster plots of cortical neurons (4th row), along with an extracted tilt angle profile (1st row). The phase portraits in the bottom row illustrate the trajectories of these individual fall-recovery cycles. Color codes for segments represent the three choices of actions as in Figures 4 and 5. Dotted curves below the tilt angle profile represent the time series data of absolute value |θ(t)−ε| where *ε* = 0.001 (the offset) used for the cycle extraction.

To characterize the model-generated network dynamics associated with sway-locked beta modulation, we examined the model-generated internal neural dynamics of the CBGT network (Fig. 5). It should be emphasized that while body sway kinematics $\left(\theta ,\dot{\theta }\right)$ and scalp EEG beta modulations are directly validated against empirical human postural data, the subcortical population firing rates and striatal LFP patterns shown in Figures 5 and 6 represent computational model predictions that provide testable hypotheses for future neurophysiological investigations.

In both intermittent and forced-choice continuous-like control models, the alternation between forward and backward active torques was closely coupled with reciprocal firing rate modulations within the motor cortex (Fig. 5*A*,*B*, top and second rows). Specifically, active torques were executed when the spike rates of the corresponding cortical populations reached the 50 Hz decision threshold. However, a fundamental difference emerged in the temporal structure of these neural commands. The intermittent control model displayed clear, prolonged intervals during which neither the forward nor backward cortical population reached the threshold (Fig. 5*A*, top and second rows). These gaps successfully defined the control-off DT. In contrast, the forced-choice continuous-like control model exhibited immediate, antagonistic transitions: as soon as one population dropped below the threshold, the other crossed it without delay (Fig. 5*B*, top and second rows).

This difference in network operation profoundly impacted the simulated LFPs in the motor cortex (Fig. 5*A*,*B*, third row). The intermittent model-generated robust, phasic LFP modulations locked to the postural sway cycle. Conversely, the forced-choice continuous-like model-generated tonic, unmodulated LFP activity, where the amplitude of sway-coupled modulation was markedly attenuated (Fig. 5, right). This loss of phasic complexity was further reflected in the population-wide raster plots (Fig. 5*A*,*B*, bottom panels). While the intermittent network sustained high-complexity, structured firing patterns across all nuclei, the forced-choice network fell into stereotyped, simple alterations between antagonistic populations.

To better capture these dynamics at a single-action resolution, we isolated individual fall-recovery cycles (Fig. 6). In the intermittent model, each cycle began with a hyperbolic segment near the upright position during the control-off DT phase (black), followed by a clockwise arc driven by a discrete burst of backward torque (blue; Fig. 6*A*, phase portrait). This sequence was supported by well-timed, transient escalations in cortical firing rates and discrete LFP bursts (Fig. 6*A*, temporal traces). Conversely, the fall-recovery cycle in the forced-choice model consisted of transitions directly from forward-torque (orange) to backward-torque actions (blue) via a negligible null-action gap (Fig. 6*B*, phase portrait). Thus, in the forced-choice continuous-like regime, the CBGT network exhibited markedly reduced temporal modulation and predominantly tonic activity.

### Post-movement beta rebound (ERS) emerges in the intermittent control regime but not in the forced-choice continuous-like regime (Experiment 1 continued)

**Figure 7.**
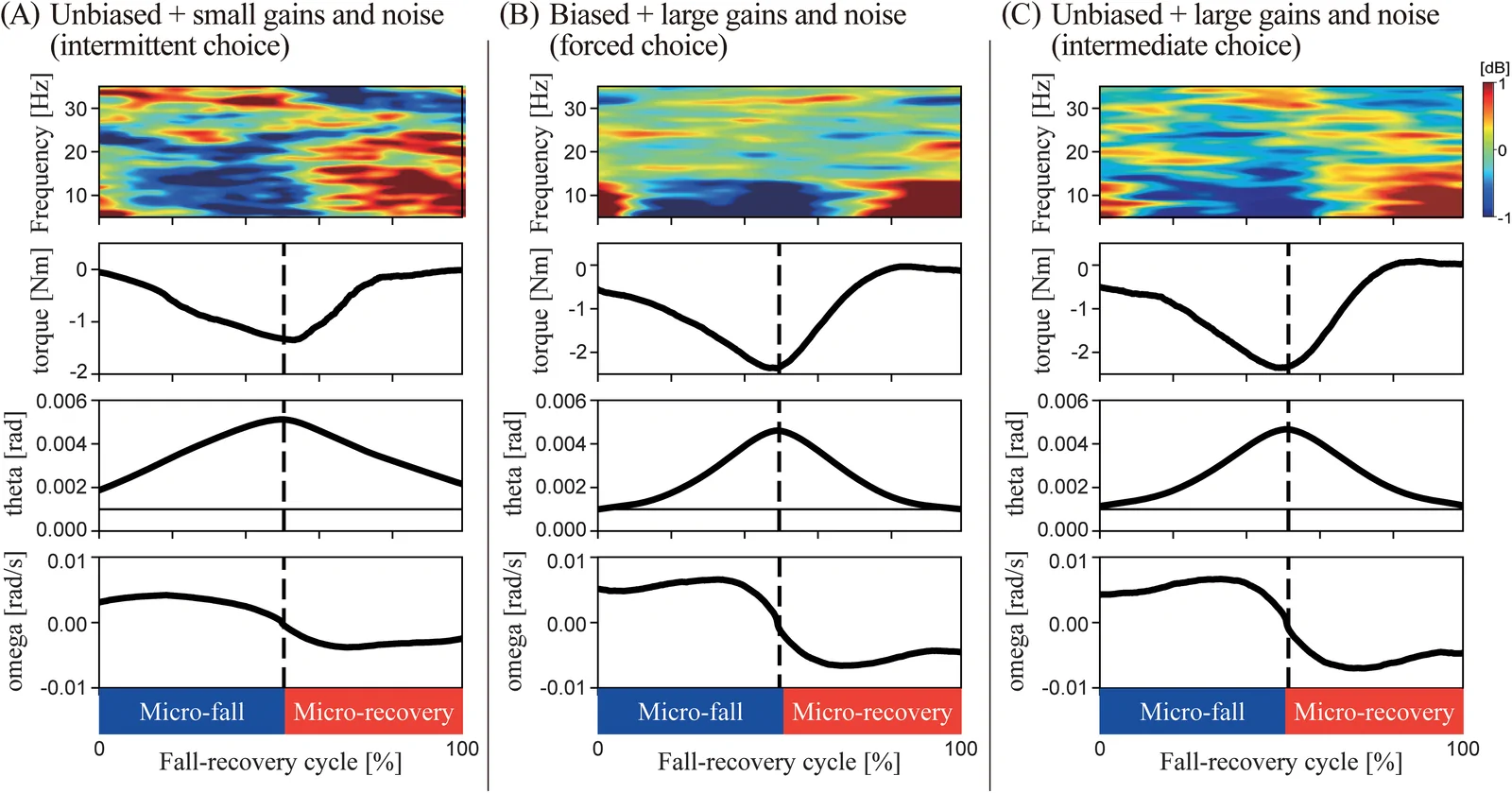
Beta ERSP locked to the micro-fall/recovery cycle for the intermittent CBGT controller, but no beta modulation for the forced-choice CBGT controller. ***A***, The intermittent control model with $W_{\mathrm{unbiased}}$ and $\left(P_{\mathrm{small}},D_{\mathrm{small}},\sigma_{\mathrm{small}}\right)$. ***B***, The forced-choice continuous-like control model with $W_{\mathrm{biased}}$ and $\left(P_{\mathrm{large}},D_{\mathrm{large}},\sigma_{\mathrm{large}}\right)$. ***C***, A type of intermediate-choice model with $W_{\mathrm{unbiased}}$ and $\left(P_{\mathrm{large}},D_{\mathrm{large}},\sigma_{\mathrm{large}}\right)$. ERSP of simulated EEG and dynamics of the pendulum averaged across all fall-recovery cycles for each control model in Experiment 1. Only forward falling cycles were used for the ensemble average. Top, Ensemble-averaged ERSPs of reconstructed LFPs over all fall-recovery cycles. Second row, Control torque applied to the pendulum. Third and 4th rows, angle and angular velocity of the pendulum, respectively. 0% and 100% in horizontal axis for all panels represent the onset of the micro-fall and offset of the micro-recovery, respectively. Vertical dashed lines represent the division between the micro-fall and the micro-recovery.

To determine whether our embodied CBGT network replicates human scalp EEG signatures, we analyzed ERSPs computed from the motor cortex LFPs during the isolated fall-recovery cycles (Fig. 7). The dynamic modulation of the beta-band (13–25 Hz) provided the pivotal finding of this study (Fig. 7, top row).

In the intermittent control model, a prominent beta-ERD occurred during the forward micro-fall phase, coinciding with the cortical preparation and execution of backward torque to arrest the pendulum (Fig. 7*A*, top). Crucially, during the subsequent micro-recovery phase—after the pendulum's velocity reversed and the active torque returned to zero (the control-off DT window)—a striking beta-ERS (or post-movement beta rebound) emerged (Fig. 7*A*, top).

In stark contrast, this beta-band rhythmicity was completely abolished in the forced-choice control model (Fig. 7*B*, top). The forced-choice continuous-like regime failed to exhibit any statistically significant beta-ERD or beta-ERS across the normalized sway cycle. Interestingly, both models successfully replicated power modulations in the lower frequency bands (theta and alpha, 5–13 Hz), showing ERD during micro-falls and ERS during micro-recoveries. In the high-frequency range (30–35 Hz), both models also captured micro-fall ERS and micro-recovery ERD, though it was less pronounced in the continuous regime. Thus, within the model, sway-phase-locked beta modulation was associated with the temporal structure of intermittent motor selection rather than with sensory input alone.

### Phasic neuronal population modulations and spike-rate profiles across the CBGT network (Experiment 1 continued)

**Figure 8.**
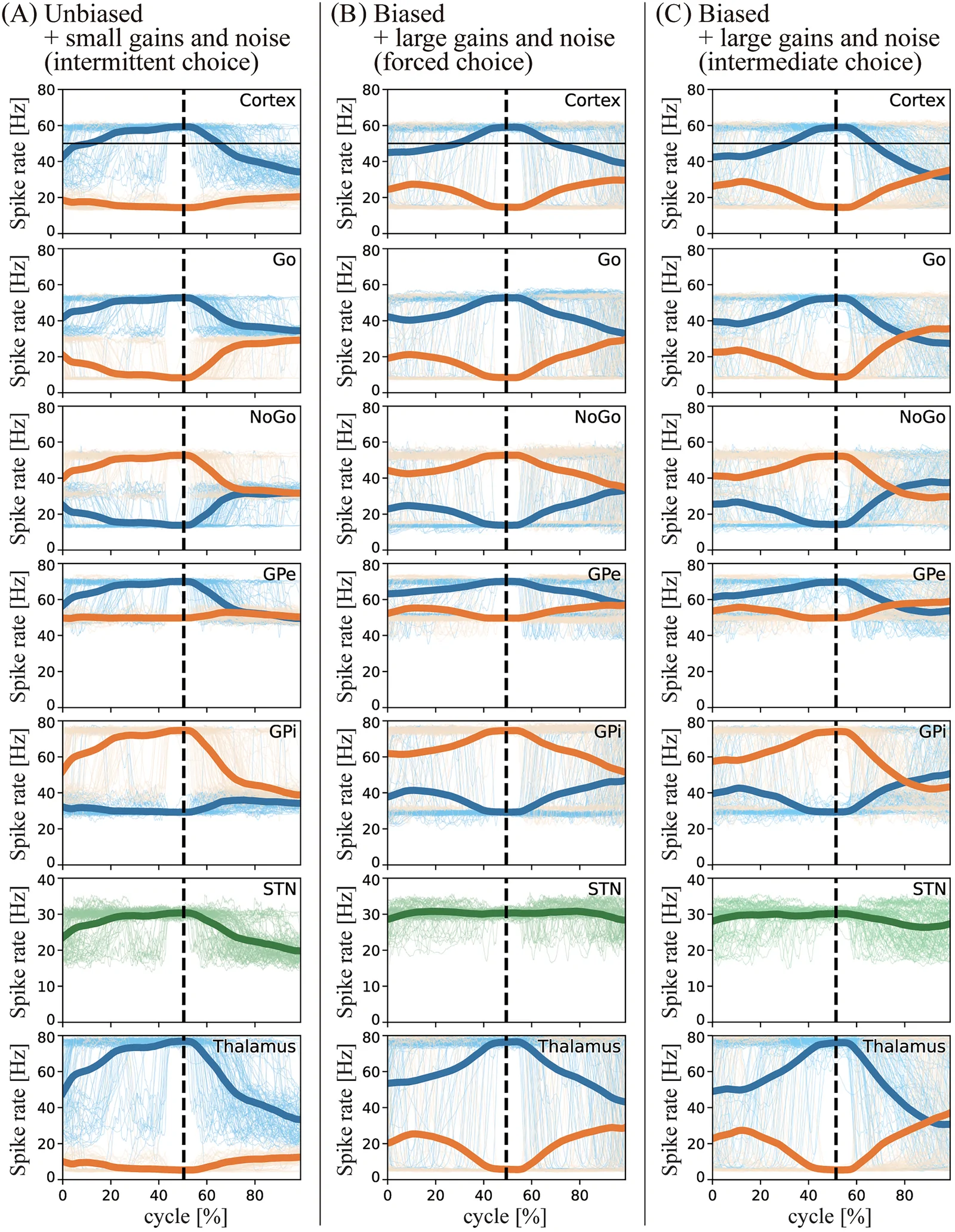
Spike rate dynamics in the CBGT controllers. ***A***, The intermittent control model with $W_{\mathrm{unbiased}}$ and $\left(P_{\mathrm{small}},D_{\mathrm{small}},\sigma_{\mathrm{small}}\right)$. ***B***, The forced-choice continuous-like control model with $W_{\mathrm{biased}}$ and $\left(P_{\mathrm{large}},D_{\mathrm{large}},\sigma_{\mathrm{large}}\right)$. ***C***, A type of intermediate-choice model with $W_{\mathrm{unbiased}}$ and $\left(P_{\mathrm{large}},D_{\mathrm{large}},\sigma_{\mathrm{large}}\right)$. Spike rate dynamics of the backward (blue) and forward (orange) populations in the cortex, Go-striatum, NoGo-striatum, GPe, GPi, STN, and thalamus in Experiment 1. In each panel, thin curves represent the spike rates of 100 single cycles, and thick curves represent the spike rates of the ensemble average of all cycles.

To characterize the population dynamics associated with beta-ERD and beta-ERS in the simulated LFP shown in Figure 7, we tracked the ensemble firing rates of the backward and forward populations within each CBGT nucleus across the fall-recovery cycle (Fig. 8).

In the intermittent control model (Fig. 8*A*), the temporal evolution of cortical spike rates was closely coupled with the postural sway cycle. During the forward micro-fall phase, the spike rate of the backward-action population was elevated, remaining near or above the 50 Hz decision threshold, whereas it decreased to ∼30–35 Hz during the subsequent micro-recovery phase. Conversely, the spike rate of the forward-action population remained low at ∼20 Hz throughout the entire fall-recovery cycle.

The cortical LFP is defined as the summation of PSCs across all cortical neurons. These PSCs integrate inputs from the thalamocortical projections, a continuous 100 Hz sensory feedback signal, and reciprocal inhibition between the two antagonistic cortical populations. Importantly, the frequency components of the LFP strongly reflect the underlying spike rates of all cortical and thalamic neurons.

These cortical spike rates were heavily influenced by excitatory projections from the thalamus; indeed, the spike-rate modulations of the two thalamic populations were qualitatively identical to those observed in the cortex. When evaluating the frequency components of the LFP based on the mean spike rates of both the backward and forward populations across the cortex and thalamus, the aggregate spike rate averaged ∼35–40 Hz during the micro-fall phase and decreased to 20–25 Hz during the micro-recovery phase. This attenuation in the aggregate firing rate was associated with increased beta-band power in the cortical LFP during the micro-recovery phase, corresponding to the post-movement beta-ERS.

Furthermore, the spike rates of the two populations in the GPi, which provides inhibitory output to the thalamus, exhibited modulations that mirrored the thalamocortical activity, albeit with the backward and forward populations reversed. Specifically, during the micro-fall phase, the spike rate of the forward population in the GPi was high and subsequently decreased during the micro-recovery phase, while the backward GPi population maintained a nearly constant low spike rate throughout the cycle. The spike-rate changes in the two GPe populations, which inhibit the GPi, mirrored the modulations of the GPi.

A prominent characteristic shared across the cortex, thalamus, GPi, and GPe was that the spike rates of individual populations, as well as the aggregate firing rates of both populations within each nucleus, exhibited clear phasic modulations coupled with the fall-recovery cycle. These phasic dynamics were closely associated with STN activity, which provides excitatory projections to both the GPe and GPi and exhibited robust phasic spike-rate modulations (see the results for the STN-dissected experiments described below in Figs. 10, 11).

In contrast, in the forced-choice control model (Fig. 8*B*), the cortical spike rates followed a distinct and less modulated temporal profile. During the forward micro-fall phase, the backward population maintained a persistently high spike rate near the 50 Hz decision threshold, peaking at ∼60 Hz near the transition from the fall to the recovery phase, and remained similarly elevated during the subsequent micro-recovery phase. Meanwhile, the spike rate of the forward population mirrored the temporal profile of the backward population around a horizontal baseline of ∼35 Hz. Consequently, the mean aggregate spike rate of the combined backward and forward cortical populations remained nearly constant at ∼35 Hz throughout the entire fall-recovery cycle. Because the spike-rate modulations of the two thalamic populations were qualitatively identical to those of the cortex, no beta-band power modulation occurred.

The spike-rate profiles in all other nuclei of the forced-choice control model were qualitatively similar to those observed in the cortex. Notably, the STN spike rate remained nearly constant throughout the fall-recovery cycle, exhibiting enhanced tonic spiking activity fixed at ∼30 Hz.

### Decoupling synaptic weights and mechanical dynamics: beta modulation as an emergent closed-loop property

**Figure 9.**
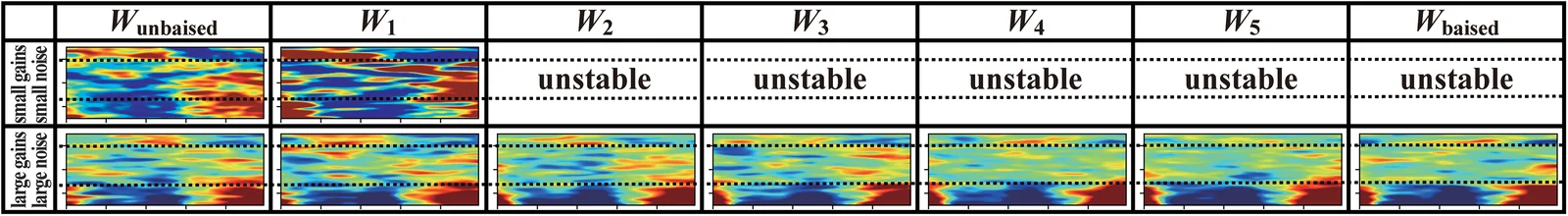
Sensitivity analysis of corticostriatal weights and mechanical gains. ERSP was examined for varied corticostriatal weight vector W ranging between $W_{\mathrm{unbiased}}$ and $W_{\mathrm{biased}}$ across five equally spaced intermediate points $W_{1}$ (close to $W_{\mathrm{unbiased}}$) to $W_{5}$ (close to $W_{\mathrm{biased}}$). Top row, Under the flexible mechanical regime with small gains and noise $\left(P_{\mathrm{small}},D_{\mathrm{small}},\sigma_{\mathrm{small}}\right)$. Bottom row, Under the rigid mechanical regime with large gains and noise $\left(P_{\mathrm{large}},D_{\mathrm{large}},\sigma_{\mathrm{large}}\right)$. Stability was achieved exclusively with $W_{\mathrm{unbiased}}$ and $W_{1}$ for $\left(P_{\mathrm{small}},D_{\mathrm{small}},\sigma_{\mathrm{small}}\right)$. For $\left(P_{\mathrm{large}},D_{\mathrm{large}},\sigma_{\mathrm{large}}\right)$, sway-related beta modulation vanished for all examined weight vectors, even when $W_{\mathrm{unbiased}}$ was preserved but combined with large mechanical gains and noise $\left(P_{\mathrm{large}},D_{\mathrm{large}},\sigma_{\mathrm{large}}\right)$.

To address whether the observed sway-related beta modulation is an artifact manually hard-coded via the corticostriatal weight matrix $\left(W_{\mathrm{unbiased}}\right)$ or an emergent property of closed-loop body–brain dynamics, we conducted systematic sensitivity analyses and decoupled the neural synaptic weights from the mechanical feedback gains (Figs. 4*C*–8*C*, 9).

#### Postural stability as a criterion for weight selection:

We first interpolated the eight-dimensional corticostriatal weight vector W between $W_{\mathrm{unbiased}}$ (s=0) and $W_{\mathrm{biased}}(s=1)$ across five intermediate points ( $W_{1}$ to $W_{5}$; s=1/6,…,5/6). Under the healthy mechanical regime with small gains and noise $\left(P_{\mathrm{small}},D_{\mathrm{small}},\sigma_{\mathrm{small}}\right)$, successful stabilization of the inverted pendulum was achieved exclusively with $W_{\mathrm{unbiased}}$ and $W_{1}$ (Fig. 9). All other weight configurations ( $W_{2}$ through $W_{5}$ and $W_{\mathrm{biased}}$) failed to maintain upright stance, leading to mechanical falling (Fig. 9). This demonstrates that $W_{\mathrm{unbiased}}$ was constrained a priori by the functional requirement of postural balance, rather than tuned a posteriori to reproduce beta modulation.

#### Quenching of beta modulation under high sway velocity:

Crucially, when $W_{\mathrm{unbiased}}$ was preserved but combined with large mechanical gains and noise $\left(P_{\mathrm{large}},D_{\mathrm{large}},\sigma_{\mathrm{large}}\right)$, sway-related beta modulation completely vanished (Fig. 7*C*), and the system yielded dynamics virtually indistinguishable from the forced-choice model (Figs. 4*C*–8*C*, 9). Although $W_{\mathrm{unbiased}}$ provided balanced Go/NoGo inputs in safe phase regions $\left(S_{2},S_{4}\right)$, the high angular velocity driven by large *P*-*D* gains forced the body's sway state to rapidly pass through $S_{2}$ and $S_{4}$. Consequently, cortical firing rates rapidly crossed the 50 Hz threshold, depriving the CBGT circuit of sufficient physical time to maintain prolonged DT (Figs. 5*C*, 6*C*, 8*C*). Without sustained Go/NoGo competitive dynamics, the circuit failed to generate the LFP spectral fluctuations required for beta-ERD/ERS (Fig. 7*C*).

These results show that sway-related beta modulation is not a built-in consequence of the corticostriatal weight configuration alone but emerges in the model when balanced CBGT dynamics and moderate physical sway velocities are appropriately matched.

### Structural dissection and parameter manipulations identify circuit dependencies of LFP frequency modulations (Experiment 2)

**Figure 10.**
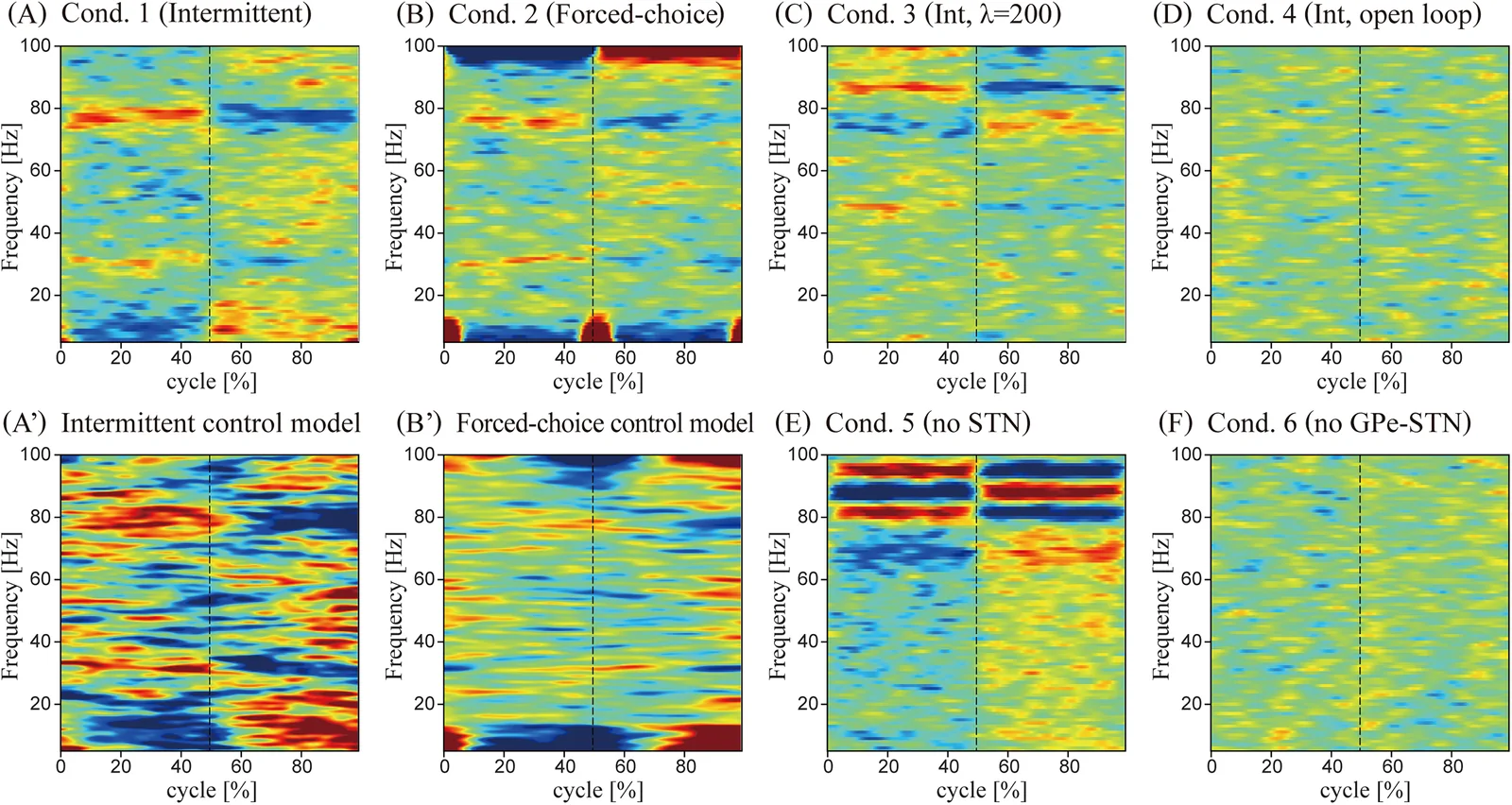
Structural dissections and hyper-sensory drive abolish or alter sway-locked cortical beta modulations. Ensemble-averaged ERSP of simulated EEG in Experiment 2 with four conditions. For all conditions, the pendulum moved passively between the regions *S*_1_ and *S*_4_ defined in Figure 2 according to a periodic prescribed trajectory. ***A***, Condition 1 with the intermittent CBGT controller. ***B***, Condition 2 with the forced-choice CBGT controller. ***C***, Condition 3 for a high spike rate of the sensory cortex with the intermittent CBGT controller. ***D***, Condition 4, where the closed-loop projection from the thalamus to the cortex was severed for the intermittent CBGT controller. ***E***, Condition 5, where the projection from the cortex to the STN was severed to prevent STN neurons from spiking for the intermittent CBGT controller. ***F***, Condition 6, where the local inhibitory projection from GPe to STN was severed for the intermittent CBGT controller. ***A***′, ***B***′, ERSPs of the intermittent and forced-choice control models (Fig. 7*A*,*B*) are redisplayed, respectively, as (***A***′) and (***B***′) with the upper frequency limit extended to 100 Hz.

**Figure 11.**
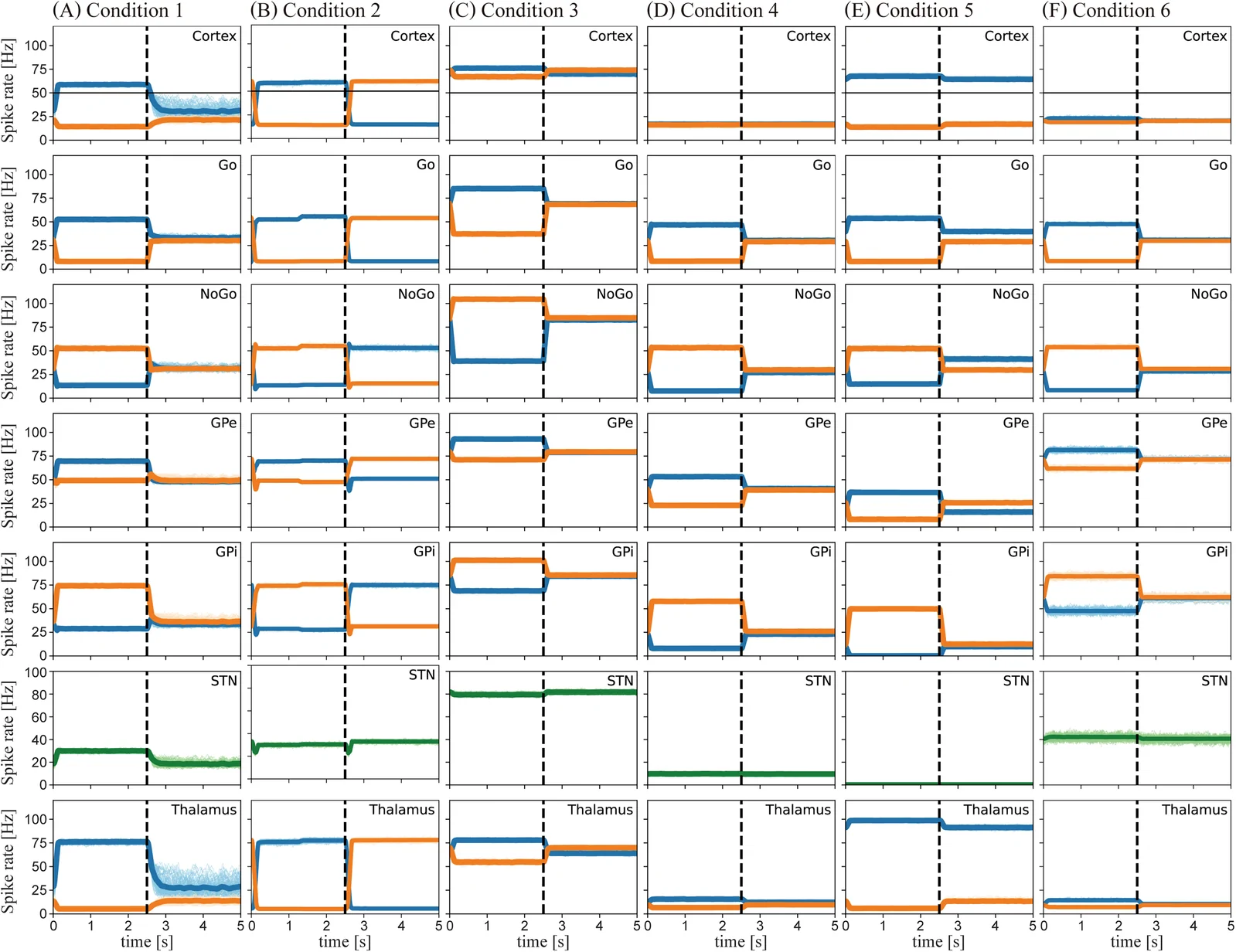
Spike rates of each neuronal population in Experiment 2. From the top to the bottom, spike rates of cortex, Go-striatum, NoGo-striatum, GPe, GPi, STN, and thalamus are shown. Blue and orange lines represent the spike rates of the backward and forward populations, respectively. Thin curves represent the spike rates of 100 single cycles, and thick curves represent the spike rates of the ensemble average of all cycles. ***A***, Condition 1. ***B***, Condition 2. ***C***, Condition 3. ***D***, Condition 4. ***E***, Condition 5. ***F***, Condition 6.

In Experiment 2, to determine how specific circuit components and parameter manipulations affect sway-related beta modulation within the model, we systematically analyzed how specific parameter manipulations and circuit lesions altered both the ERSPs (Fig. 10) and the corresponding population-specific spike rates across each nucleus (Fig. 11). To isolate intrinsic circuit dynamics from the confounding effects of active postural stabilization, the inverted pendulum was moved passively along predefined periodic trajectories. For this comprehensive spectral analysis, the frequency range of the ERSP was extended up to 100 Hz. For baseline comparison, the ERSPs of the intermittent and forced-choice control models originally shown in Figure 7 are replotted with this expanded 100 Hz frequency limit in Figure 10*A*′,*B*′, respectively.

First, we examined Condition 1, in which the pendulum was driven along a passive, periodic trajectory designed to mimic a typical sway profile of the intermittent control model, utilizing the original parameter set of the intermittent CBGT controller (Figs. 10*A*, 11*A*). Although the overall modulation amplitudes of ERD and ERS across individual frequency bands were slightly attenuated (Fig. 10*A*), the resulting ERSP profile was qualitatively identical to that of the active intermittent control model (Fig. 10*A*′). Furthermore, the movement-cycle dependence of the spike rates in each nucleus (Fig. 11*A*) remained quantitatively consistent with the active control results (Fig. 8*A*).

Focusing on the high-frequency range (>50 Hz), which was not detailed in Figure 7, both Figure 10*A* and *B*′ revealed a prominent power modulation ∼80 Hz, characterized by an ERS in the first half of the cycle followed by an ERD in the second half. Additionally, weaker power modulations—consisting of an ERD in the first half and an ERS in the second half—were observed in the adjacent bands above (90–100 Hz) and below (50–70 Hz) this central range. Based on the spike-rate profiles (Fig. 11*A*), the 80 Hz ERS during the first half of the cycle closely corresponds to the aggregate (summed) spike rates, rather than the simple mean, of the backward and forward populations within the cortex and thalamus. Similarly, the subtle ERS in the 50–70 Hz range during the second half of the cycle reflects the aggregate spike rate of these two antagonistic populations during that specific period.

In Condition 2, the pendulum was driven along a passive trajectory mimicking one cycle of the forced-choice control model, utilizing the continuous CBGT parameters (Figs. 10*B*, 11*B*). The beta-band ERSP obtained in Condition 2 closely matched that of the active continuous model (Fig. 10*B*′), displaying a complete absence of power modulation. Minor discrepancies in the alpha and theta bands between the active and passive conditions were attributable to differences between the unconstrained organic trajectories in Experiment 1 and the prescribed cyclic trajectory in Experiment 2. This tonic, unmodulated beta power was accompanied by stable spike rates across all nuclei throughout the movement cycle (Fig. 11*B*), consistent with Figure 8*B*.

Intriguingly, in both Figure 10*B* and *B*′, phasic modulations emerged in the higher frequency ranges: a distinct 80 Hz rhythm showed an ERS in the first half and an ERD in the second half of the cycle, whereas a 100 Hz rhythm displayed a reversed pattern (an ERD in the first half and an ERS in the second half). Furthermore, smaller-amplitude modulations with an ERS in both the first and second halves emerged ∼30 Hz. Crucially, these specific power modulations lacked clear corresponding spike-rate modulations in Figure 11*B*, whether evaluating individual, mean, or aggregate population firing rates. This mismatch strongly implies that these high-frequency spectral profiles are emergent phenomena resulting from the intrinsic closed-loop dynamics of the CBGT architecture.

In Condition 3, where the sensory cortex spike rate representing the pendulum state was doubled (*λ* = 200 Hz), the characteristic beta-band power modulation was completely abolished (Figs. 10*C*, 11*C*). The 35 Hz ERS in the first half of the cycle and its subsequent ERD, which were prominent in Condition 1, vanished under this hyper-sensory drive. Instead, the most dominant power modulation shifted to the 80–90 Hz range. This shifted profile consisted of a sub-80 Hz modulation (an ERD in the first half and an ERS in the second half) and adjacent modulations just below and above 80 Hz (characterized by an ERS in the first half and an ERD in the second half, respectively). Although the cortical and thalamic spike rates (Fig. 11*C*) did not exhibit modulations that directly mirrored these specific frequency boundaries, they appeared to align with the individual population spike rates hovering ∼75 Hz, which diverged between the first and second halves of the cycle. However, similar to the findings in Condition 2, these complex spectral signatures represent emergent properties of the closed-loop CBGT circuit dynamics rather than simple linear reflections of firing rates.

In Condition 4, the closed-loop thalamocortical projection was severed in the intermittent CBGT configuration (Figs. 10*D*, 11*D*). Following this disruption, power modulations were completely eliminated across all frequency bands (Fig. 10*D*). Under this open-loop condition, the cortical spike rates for both the backward and forward populations remained fixed at a tonic baseline of ∼18 Hz (Fig. 11*D*). This finding shows that, within the model, the rhythmic power modulations require an intact thalamocortical component of the CBGT closed-loop.

In Condition 5, the hyperdirect projection from the cortex to the STN was severed to suppress STN spiking activity within the intermittent CBGT framework (Figs. 10*E*, 11*E*). Under this condition, the ERSP displayed a broad but weak power modulation stretching from 10 to 60 Hz, characterized by a diffuse transition from an ERD in the first half to an ERS in the second half of the cycle. In the high-frequency range, robust power modulations emerged across four distinct, sharply defined bands centered ∼70, 80, 90, and 100 Hz. These bands exhibited sequential transitions of ERD-to-ERS, ERS-to-ERD, ERD-to-ERS, and ERS-to-ERD, respectively (Fig. 10*E*). Because the mean spike rates of the backward and forward populations in both the cortex and thalamus remained constant throughout the movement cycle (Fig. 11*E*), these spectral shifts cannot be explained by fluctuations in mean firing rates. Instead, these individual high-frequency modulations likely reflect the unmasked firing rates of independent populations encoded within the LFP components. Specifically, the spike rate of the thalamic backward population was ∼100 Hz in the first half of the cycle and dropped to 90 Hz in the second half, a shift that closely aligns with the ERS observed in those respective bands. Additionally, the prominent 80 Hz ERS during the first half of the cycle appears to be associated with the combined activity of the cortical and thalamic backward populations, which fired at 60 and 100 Hz, respectively.

Finally, in Condition 6, the local inhibitory feedback projection from the GPe to the STN was severed within the intermittent CBGT controller (Figs. 10*F*, 11*F*). Mirroring the results of the thalamocortical lesion (Condition 4), power modulations were entirely abolished across all frequency bands (Fig. 10*F*). Cortical spike rates for both populations flattened into a static tonic state at ∼18 Hz (Fig. 11*F*). Taken together, these selective dissection results show that, within the model, sway-related LFP power modulation depends critically on both thalamocortical feedback and the GPe–STN loop; disrupting either component abolishes the modulation.

## Discussion

This study examined a circuit-level computational account of sway-phase-locked beta-band modulations during human quiet stance using an embodied SNN model of the CBGT circuit integrated with an inverted pendulum. Our closed-loop simulations demonstrate that the characteristic cortical EEG patterns observed during stance—specifically, beta-ERD during micro-falls and beta-ERS/post-movement rebound during micro-recoveries (Nakamura et al., 2023)—emerge in the model under an intermittent control regime. In contrast, a “forced-choice continuous-like” configuration $\left(W_{\mathrm{biased}}\right)$, characterized by unremitting active feedback without null-action DT intervals, failed to regenerate these dynamic beta modulations. Structural dissections further revealed that, within the model, these sway-coupled modulations depend critically on intact thalamocortical interactions and the GPe–STN loop, together with state-dependent sensory feedback. These findings provide a computational account linking sway-related beta dynamics to intermittent motor selection within the proposed CBGT framework.

### Noncircular emergence of beta modulation via embodied body–brain coupling

A primary concern in neuro-computational modeling is whether observed oscillations are artificially hard-coded into model parameters. Our parameter sensitivity analyses address this potential circularity. If beta-ERD/ERS were a trivial artifact of $W_{\mathrm{unbiased}}$, they should have persisted regardless of physical feedback parameters. However, coupling $W_{\mathrm{unbiased}}$ with large mechanical gains and high noise erased beta modulations. These results show that, within the model, sway-related beta modulation is not determined by striatal weight topology alone, but emerges when internal CBGT dynamics permitting prolonging of DT and physical sway dynamics are appropriately matched.

### Physiological motivation and computational abstractions

While our model uses necessary computational abstractions to execute embodied posture control, several components are motivated by established neuroanatomical and physiological observations rather than intended as literal implementations of those circuits. First, the division of motor cortical and subcortical neurons into forward- and backward-torque populations is motivated by the somatotopic parallel-channel organization of the CBGT loop (Alexander and DeLong, 1985; Hoover and Strick, 1993). Transneuronal tracing confirms discrete representation of opposing muscle groups in primates (Rathelot and Strick, 2006), where microstimulation in the putamen evokes discrete single-joint movements (Alexander and DeLong, 1985; Nambu et al., 2002). During standing, ankle stabilization relies on antagonist pairs: plantarflexors and dorsiflexors. Modeling competing torque channels therefore provides a functionally motivated abstraction of antagonist motor selection within the CBGT architecture.

Second, while the four-region phase-space partitioning $\left(S_{1}-S_{4}\right)$ abstracts multi-sensory integration (somatosensory, vestibular, visual), state-dependent corticostriatal gain modulation is well supported, as the striatum receives dense projections from sensory and motor cortices where synaptic weights dynamically encode state-action mappings. Third, the 50 Hz firing rate threshold for torque generation aligns with the accumulator/threshold model of movement initiation, supported by primate recordings showing ramping activity crossing neural threshold boundaries prior to movement (Tanaka, 2007).

### Functional relation to muscle mechanics

Our closed-loop framework is functionally motivated by division of labor among calf muscles during standing (Loram et al., 2005a; Gawthrop et al., 2011). Background stiffness maintaining ankle stance near equilibrium without excessive energy is provided by tonic soleus (SOL) activity and intrinsic tendon elasticity (∼60–90% of overturning gravitational torque). Conversely, intermittent active feedback torque generated when cortical firing rates cross the decision threshold provides an abstract representation of phasic contributions from gastrocnemius (MG) and tibialis anterior (TA). When sway trajectories reside in safe phase regions $\left(S_{2},S_{4}\right)$, active CBGT torque is suspended, leaving the system under intrinsic SOL/tendon stiffness—a model state associated with prolonged DT and beta-ERD/ERS.

### Contextualization with classical postural control frameworks

Continuous sensory-reweighting frameworks (Peterka, 2002; Mergner, 2010) and intermittent act-and-see models (Loram et al., 2005b; Asai et al., 2009; Gawthrop et al., 2011) have established how sensory integration achieves robust stabilization. Introducing a forced-choice continuous regime within the CBGT network was not designed to refute continuous models, but to serve as an internal computational contrast. By comparing forced-choice against balanced intermittent regimes, our model demonstrates how the same spiking CBGT architecture can exhibit continuous-like torque generation or discrete intermittent switching depending on its functional parameterization, and how these regimes are associated with distinct model-generated LFP dynamics.

### Relation to proposed mechanisms of beta modulation

Prominent hypotheses regarding beta (13–30 Hz) oscillations include intracortical E/I balance (Jensen et al., 2005; Sherman et al., 2016), thalamocortical resonance (Ching et al., 2010), canonical basal ganglia-thalamo-cortical (BGTC) loop reverberations (Leventhal et al., 2012; Liu et al., 2020), and inter-regional communication maintaining the sensorimotor “status quo” (Bonnefond et al., 2017; Spitzer and Haegens, 2017). Furthermore, the GPe–STN reciprocal network acts as a core micro-circuit beta resonator (Koelman and Lowery, 2019), whose pathological hypersynchrony characterizes Parkinson's disease (PD; Weinberger et al., 2006; Pavlides et al., 2015).

Our model provides a framework in which several of these proposed circuit contributions can be examined in the context of intermittent motor control. Under intermittent control, STN firing rates modulate phasically based on body physical states. Under the forced-choice continuous-like regime, STN activity remained comparatively tonic (∼30 Hz), accompanied by a loss of phase-locked beta modulation. Within the model, disrupting the cortico-STN hyperdirect pathway (Koketsu et al., 2021) substantially reorganized the spectral modulation, whereas disrupting the GPe–STN interaction (Fan et al., 2012) abolished sway-related LFP modulation. Together with the abolition observed after disrupting thalamocortical feedback, these manipulations show that the characteristic beta modulation depends critically on interactions among these circuit components within the present architecture.

### Functional balance of sensory corticostriatal projections

Within the model, balanced sensory corticostriatal synaptic weights are a key determinant of the action-selection dynamics associated with alternating beta-ERD/ERS. Sensory input encodes the instantaneous pendulum state relative to the stable manifold of upright equilibrium. Assigning balanced weights to opposing torque action populations establishes a competitive DT window during which neither motor command gains dominance, manifesting the “control-off” phase of intermittent control. Activity-dependent corticostriatal plasticity (e.g., in dorsolateral striatum) is critical for motor skill refinement (Cataldi et al., 2022), where dopaminergic plasticity alters direct/indirect pathway competition during decision-making (Dunovan et al., 2019). Implementing spike-timing-dependent plasticity (STDP) could enable self-organized acquisition of these balanced profiles.

### Clinical implications and hierarchical postural architecture

Human quiet stance has been successfully described by intermittent control models, and previous model-based studies suggest that reduced intermittency may contribute to postural instability in Parkinson's disease (Perera et al., 2018; Suzuki et al., 2020; Bao and Chen, 2024; Bao et al., 2026). Related alterations in sway-coupled beta modulation have also been reported in older individuals with chronic dizziness (Ellmers et al., 2025). These observations raise the possibility that changes in behavioral intermittency and beta-band dynamics may reflect interacting alterations in brain–body control, although the present model does not establish their physiological origin in clinical populations.

While lower-level posture tone and automatic reflexes involve brainstem, cerebellar, and spinal networks (Horak, 2006; Takakusaki, 2017), the present model focuses on a candidate contribution of CBGT-mediated action selection within this broader hierarchical architecture. The mechanical parameter regimes examined here were motivated by previous model-based characterization of postural control in healthy individuals and patients with Parkinson's disease (Suzuki et al., 2020). Altered beta-band activity and its modulation have been extensively associated with motor dysfunction in Parkinson's disease (Brown et al., 2001; Jenkinson and Brown, 2011; Moisello et al., 2015; Singh, 2018; Wu et al., 2019). Within the present model, parameter regimes producing increased mechanical stiffness and noise shortened DT, promoted more persistent active control, and reduced sway-related beta modulation. These results raise the testable possibility that alterations in behavioral intermittency and beta dynamics may become coupled through changes in brain–body interactions in Parkinson's disease, although establishing such a relationship physiologically will require direct experimental validation.

### Methodological limitations, extensibility, and future directions

An important limitation is that the present CBGT network is a functionally and anatomically motivated computational abstraction rather than a biophysically constrained reconstruction of the human postural-control circuitry. Accordingly, reproducing sway-related beta modulation demonstrates sufficiency within the proposed architecture but does not establish that the same circuit interactions generate these dynamics physiologically.

While LIF models isolate network topology interactions with physical dynamics, upgrading to conductance-based Hodgkin–Huxley or Izhikevich-type neurons would incorporate intrinsic STN/GPe burst-firing properties. Additionally, driving sensory corticostriatal weights via autonomous reinforcement learning (Dunovan et al., 2019) could simulate self-organized emergence of postural beta rhythms, since intermittent strategies natively emerge when balancing displacement against metabolic cost (Takazawa et al., 2024). Finally, assimilating our CBGT model with empirical human kinematics and high-density EEG via Bayesian inference (Suzuki et al., 2020; Yokoyama et al., 2025) will allow more direct data-driven evaluation of these model predictions.

### A candidate brain–body framework linking intermittent control and beta dynamics

Traditionally, exaggerated beta oscillations and postural instability in PD have been investigated separately. The present model offers a framework for examining how behavioral intermittency and sway-related beta dynamics may become coupled through closed-loop brain–body interactions. Within the model, beta modulation emerges when CBGT action-selection dynamics permitting prolonged DT are appropriately matched to the physical sway dynamics, whereas parameter regimes producing faster sway and shortened DT abolish this modulation. These results identify a model-level link between behavioral intermittency and beta-band dynamics and generate the testable hypothesis that alterations in their temporal coordination may contribute to postural impairment. Determining whether this relationship reflects the physiological mechanisms operating in Parkinson's disease will require direct validation using neural and behavioral data.

## Synthesis

Reviewing Editor: David Schoppik, New York University Grossman School of Medicine Decisions are customarily a result of the Reviewing Editor and the peer reviewers coming together and discussing their recommendations until a consensus is reached. When revisions are invited, a fact-based synthesis statement explaining their decision and outlining what is needed to prepare a revision will be listed below. The following reviewer(s) agreed to reveal their identity: NONE. Note: If this manuscript was transferred from JNeurosci and a decision was made to accept the manuscript without peer review, a synthesis may not be available.

The revised manuscript addresses the outstanding issues identified by the reviewers.
